# Fibroblast-Derived STC-1 Modulates Tumor-Associated Macrophages and Lung Adenocarcinoma Development

**DOI:** 10.1016/j.celrep.2020.107802

**Published:** 2020-06-23

**Authors:** Tamihiro Kamata, Tsz Y. So, Qasim Ahmed, Susan Giblett, Bipin Patel, Jinli Luo, Roger Reddel, Catrin Pritchard

**Affiliations:** 1 Leicester Cancer Research Centre, University of Leicester, Leicester Royal Infirmary, Leicester LE2 7LX, UK; 2 Department of Molecular Cell Biology, University of Leicester, Lancaster Road, Leicester LE1 9HN, UK; 3 Cancer Research Unit, Children’s Medical Research Institute, University of Sydney, Westmead, NSW, Australia

**Keywords:** lung adenocarcinoma, stanniocalcin-1, tumor-associated macrophages, tumor-associated fibroblasts, tumor microenvironment

## Abstract

The tumor microenvironment (TME) consists of different cell types, including tumor-associated macrophages (TAMs) and tumor-associated fibroblasts (TAFs). How these cells interact and contribute to lung carcinogenesis remains elusive. Using ^G12D^KRAS- and ^V600E^BRAF-driven mouse lung models, we identify the pleiotropic glycoprotein stanniocalcin-1 (STC1) as a regulator of TAM-TAF interactions. STC1 is secreted by TAFs and suppresses TAM differentiation, at least in part, by sequestering the binding of GRP94, an autocrine macrophage-differentiation-inducing factor, to its cognate scavenger receptors. The accumulation of mature TAMs in the *Stc1*-deficient lung leads to enhanced secretion of TGF-β1 and, thus, TAF accumulation in the TME. Consistent with the mouse data, in human lung adenocarcinoma, *STC1* expression is restricted to myofibroblasts, and a significant increase of naive macrophages is detected in *STC1*-high compared with *STC1*-low cases. This work increases our understanding of lung adenocarcinoma development and suggests new approaches for therapeutic targeting of the TME.

## Highlights

• STC1 is expressed and secreted from TAFs
• STC1 depletion results in an accumulation of mature TAMs and TAFs in mouse lung models
• Fibroblast-derived STC1 binds to GRP94, preventing macrophage differentiation
• STC1^high^ human lung adenocarcinomas have increased unpolarized TAMs

## Graphical Abstract

**Figure:**
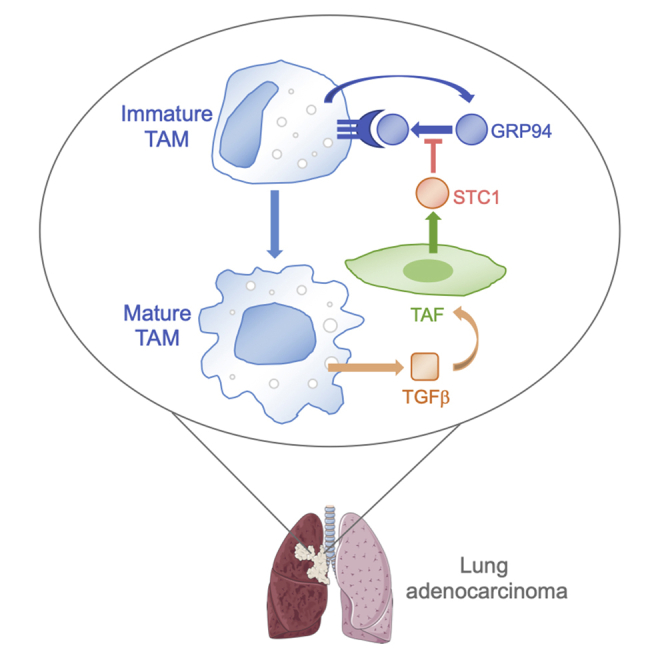

## Teaser

The tumor microenvironment contains heterogeneous cell types, but how these cells interact to regulate tumor progression is unknown. Kamata et al. identify the fibroblast-derived, secreted glycoprotein STC1 as a paracrine regulator of tumor-associated macrophage differentiation in lung adenocarcinoma.

## Introduction

The tumor microenvironment (TME) consists of heterogeneous non-malignant cell types, including inflammatory immune cells, tumor-associated fibroblasts (TAFs), and angiogenic vascular cells in the extracellular matrix (ECM)-rich stroma (Lu et al., 2012, Quail and Joyce, 2013). It is believed that the TME has an integral role in tumor progression and that collaborative interactions not only between tumor cells and the TME but also among different cell types within the TME contribute to optimized remodeling of the TME to support tumor progression (Hanahan and Coussens, 2012, Palucka and Coussens, 2016). However, it remains elusive as to how the different cell types within the TME interact with each other, although secreted factors are thought to have a role, at least in part.

We have focused our investigations on identifying secreted factors that have a role in regulating TME-tumor interactions during lung adenocarcinoma development. Our secretome analysis using the ^V600E^BRAF-driven mouse lung adenoma model identified stanniocalcin-1 (STC1) as a candidate mediator of tumor-TME interactions (Kamata et al., 2015). Mammalian STC1 is a secreted glycoprotein suggested to function as a local autocrine/paracrine factor involved in calcium homeostasis and oxidative stress responses (Yeung et al., 2012). The paracrine functions of STC1 are thought to be mediated through internalization of the protein into target cells, followed by transfer to the mitochondria where STC1 regulates superoxide generation (Ohkouchi et al., 2012, Wang et al., 2009). Despite this, *Stc1*-deficient mice do not show an obvious phenotype, suggesting STC1 is largely redundant under physiological conditions (Chang et al., 2005).

It is well-recognized that *STC1* expression is deregulated in human cancers. High *STC1* expression is associated with poor prognosis in some, but not all, cancers (Chang et al., 2015, Shirakawa et al., 2012, Su et al., 2015, Tamura et al., 2011, Yeung et al., 2015). In the context of tumor-TME interactions, *STC1* was reported as being upregulated in breast cancer-educated fibroblasts, but no *in vivo* effects of fibroblast-derived STC1 in co-xenotransplantation experiments were identified (Rajaram et al., 2013). In contrast, orthotopic xenotransplantation of *Stc1*-deficient fibroblasts with human colon cancer cells led to reduced metastasis (Peña et al., 2013), suggesting fibroblast-derived STC1 contributes to cancer progression. The human *STC1* gene is located on the short arm of chromosome 8, a region frequently deleted in lung adenocarcinoma (Weir et al., 2007), but any tumor-suppressor functions of STC1 in this cancer type has not yet been examined.

To investigate the role of STC1 in lung adenoma/adenocarcinoma progression, we have analyzed two genetically engineered mouse (GEM) models: one driven by ^G12D^KRAS leading to adenocarcinoma development (Sutherland et al., 2014), and the other by ^V600E^BRAF generating pre-malignant adenomas (Kamata et al., 2015). We have also investigated STC1 expression in human lung adenocarcinoma. Our data confirm STC1 as a secreted protein, derived from lung fibroblasts, which regulates tumor-associated macrophage (TAM) differentiation and TAF accumulation in the TME.

## Results

### *Stc1* Deficiency Promotes TAM/TAF Accumulation and Tumor Progression in the ^G12D^KRAS-Driven Lung Tumor Model

**Figure 1:**
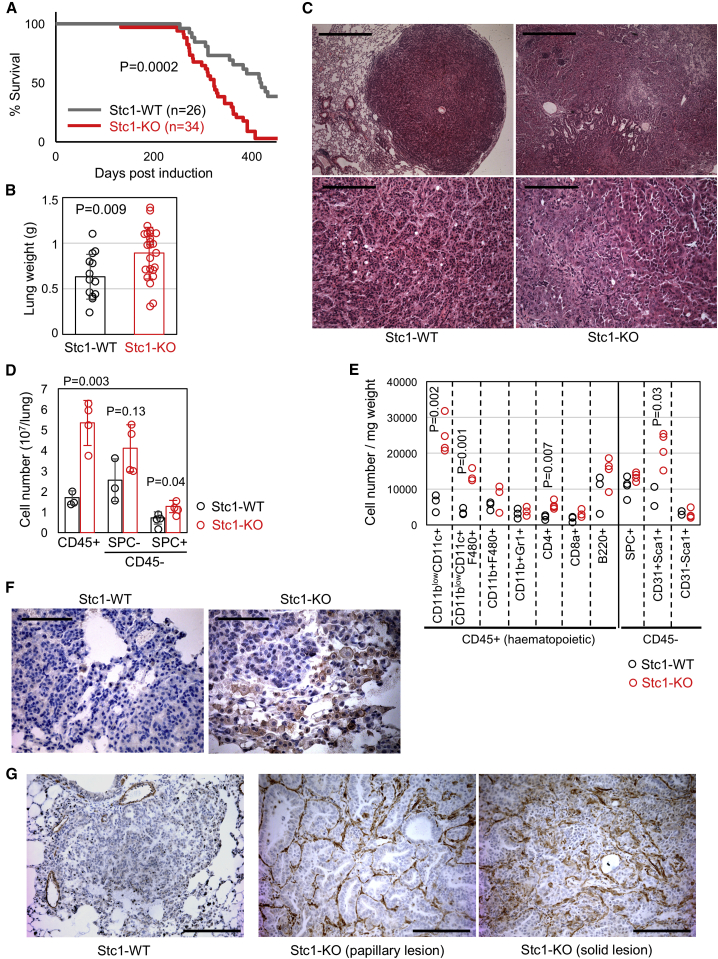
Characterization of *Stc1*^*−/−*^ SPK Mice (A) Shortened survival of *Stc1*^*−/−*^ SPK mice (Stc1-knockout [Stc1-KO]) compared with *Stc1*^*+/+*^ counterparts (Stc1-wild type [WT]). (B) Increased lung weights of *Stc1*^*−/−*^ SPK mice (Stc1-KO, n = 24) compared with *Stc1*^*+/+*^ counterparts (Stc1-WT, n = 14) at 9–13 months after induction. (C) Histological analysis of lung tumors developing in Stc1-WT/KO SPK mice at 9 months after induction. Scale bars, 500 μm (top) or 125 μm (bottom). (D) Quantitation of CD45^+^ hematopoietic, CD45^−^SPC^−^ non-hematopoietic, and CD45^−^SPC^+^ tumor/AT2 cell numbers in Stc1-WT/KO SPK lungs at 9 months after induction (n = 3–4). (E) Quantitation of myelo-lymphoid lineages within the CD45^+^ population and endothelial/mesenchymal lineages within the CD45^−^ population in Stc1-WT/KO SPK lung at 9 months after induction (n = 3–4). The cell number in each lineage is expressed relative to the lung tissue weight. (F) F4/80 immunohistochemistry of peri-tumor stroma in Stc1-WT/KO SPK lung. Scale bars, 62.5 μm. (G) αSMA immunohistochemistry of Stc1-WT/KO SPK lung sections. αSMA^+^ staining in papillary lesions (middle) and in a solid lesion (right) is shown for the Stc1-KO lung. Scale bars, 125 μm.

To investigate the *in vivo* functions of STC1 in lung tumorigenesis, we infected *Kras*^*+/LSL-G12D*^ mice on the *Stc1*^*+/+*^ and *Stc1*^*−/−*^ backgrounds with the Ad5-mSPC-Cre adenoviral vector, which allows expression of Cre recombinase from the surfactant protein C (SPC) promoter in alveolar type 2 (AT2) cells (Sutherland et al., 2014) (referred to as SPK mice hereafter). *Stc1*^*+/+*^ SPK mice started to show respiratory symptoms at 9 months after induction, and ∼50% of animals died within 400 days (Figure 1A). In contrast, most *Stc1*^*−/−*^ SPK mice died during this period (Figure 1A) and had increased lung weights compared with *Stc1*^*+/+*^ SPK mice (Figure 1B). Histological analysis showed that *Stc1*^*+/+*^ SPK tumors retained characteristics of papillary adenomas with mild to moderate dysplasia, whereas *Stc1*^*−/−*^ SPK tumors occasionally showed malignant progression to adenocarcinoma (Figure 1C). There was also evidence for extensive remodeling of the TME in the *Stc1*^*−/−*^ SPK lungs (Figure 1C).

To investigate the cellular basis for this phenotype, we performed flow cytometry quantitation (Figures 1D–1E and S1). This analysis demonstrated an increase in the number of SPC^+^ cells that mainly represent tumor cells derived from AT2 cells in the *Stc1*^*−/−*^ SPK lung (Figure 1D), although this difference was not significant when adjusted for lung weight (Figure 1E), reflecting the close relationship between tumor burden and lung weight. Interestingly, there were robust increases of stromal hematopoietic (CD45^+^) cells in the *Stc1*^*−/−*^ SPK lung (Figure 1D). Notably, CD45^+^CD11b^low^CD11c^+^ cells containing F4/80^+^ and major histocompatibility complex class II (MHCII)^+^ populations (Figure 1E and S2A), which are consistent with a TAM phenotype (Franklin et al., 2014), were significantly increased, even after adjustment for lung weight. Significant increases of CD4^+^ T cells and CD45^−^CD31^+^Sca1^+^ endothelial cells (Kotton et al., 2003) were also observed (Figure 1E).

The CD11b^low^CD11c^+^ cells in the SPK lungs were negative for dendritic cell (DC) markers CD103, CCR7, and c-Kit (Miller et al., 2012) but expressed the alveolar macrophage (AM) marker Siglec-F (Misharin et al., 2013) (Figure S2A). This supports their macrophage nature but suggests they are distinct from resident interstitial macrophages (IMs) and IM-derived TAMs, which lack Siglec-F expression (Loyher et al., 2018). Based on these findings, together with the previous report of F4/80 upregulation during TAM differentiation from monocytes recruited to the TME (Franklin et al., 2014), we define the F4/80^−^ and F4/80^+^ sub-populations in CD11b^low^CD11c^+^ cells as IMCs (immature macrophage-lineage cells) and TAMs, respectively (Figure S1A). Increased peri-tumor distribution of F4/80^+^ cells in the *Stc1*^*−/−*^ SPK lung was confirmed by immunohistochemistry (Figure 1F).

Apart from hemopoietic populations, because of the pathology observed in histological sections (Figure 1C), we were interested in assessing the TAF population. Because a robust surface marker has not been identified for flow cytometry quantitation of this cell population, we performed immunostaining for α-smooth muscle actin (αSMA), a well-established marker for TAFs (Gascard and Tlsty, 2016). This analysis showed amplification of tumor-associated αSMA^+^ cells in the fibrovascular cores (FVCs) of papillary lesions in the *Stc1*^*−/−*^ SPK lung, which sometimes extended diffusely into solid lesions (Figure 1G).

### *Stc1* Deficiency Accelerates TAM Differentiation and TAF Accumulation in the ^V600E^BRAF-Driven Lung Tumor Model

**Figure 2:**
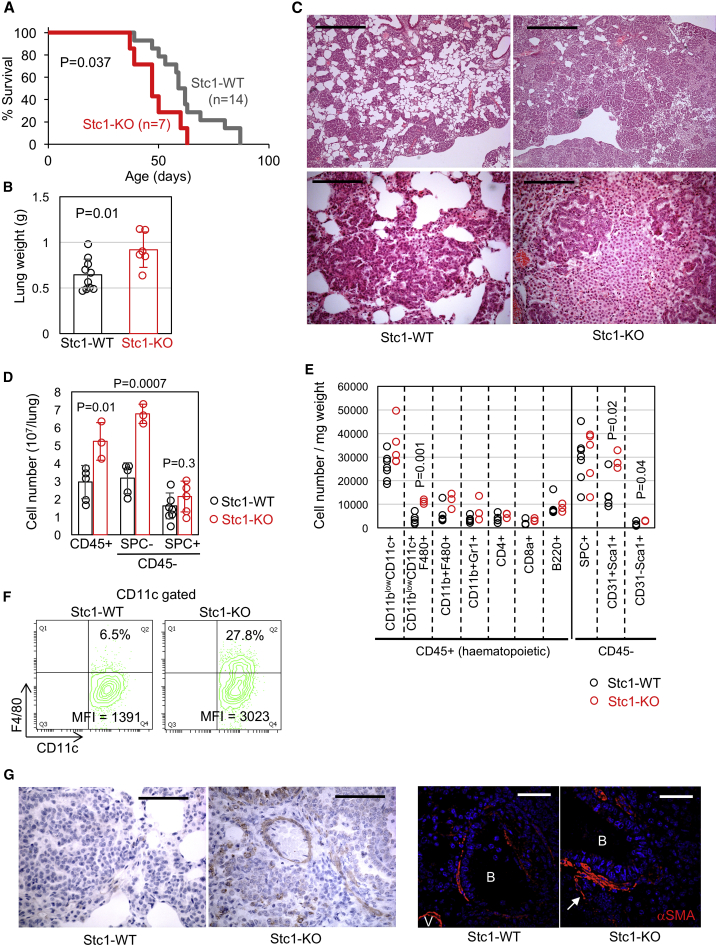
Characterization of *Stc1*^*−/−*^ BVE Mice (A) Shortened survival of *Stc1*^*−/−*^ BVE mice (Stc1-KO) compared with *Stc1*^*+/+*^ BVE mice (Stc1-WT). (B) Increased lung weights of *Stc1*^*−/−*^ BVE mice (Stc1-KO, n = 6,) compared with *Stc1*^*+/+*^ counterparts (Stc1-WT, n = 10). (C) H&E staining of lung sections from 6 wk-old Stc1-WT/KO (bottom) BVE mice. Scale bars, 500 μm (top) or 125 μm (bottom). (D) Quantitation of CD45^+^ hematopoietic, CD45^−^SPC- non-hematopoietic and CD45^−^SPC^+^ tumor/AT2 cell numbers in Stc1-WT/KO BVE lungs at 6 weeks of age (n = 3–5). (E) Quantitation of myelo-lymphoid lineages within the CD45^+^ population and endothelial/mesenchymal lineages within the CD45^−^ non-hematopoietic population in Stc1-WT/KO BVE lungs at 6 weeks of age (n = 3–7). The cell number in each lineage is expressed relative to lung tissue weight. (F) Representative flow cytometry plots for F4/80 expression on CD11c^+^ cells in Stc1-WT/KO BVE lungs. %F4/80^+^ and F4/80 mean fluorescence intensity (MFI) are indicated. (G) αSMA immunostaining of lung sections from Stc1-WT/KO BVE mice, detected by IHC (left) or confocal imaging (right). V, vessels; B, bronchioles. Arrows indicate migration of hyperplastic αSMA^+^ cells into stromal areas. Scale bars, 62.5 μm (left) or 50 μm (right).

We next asked whether a similar phenotype could be detected in the ^V600E^BRAF-driven lung model. To this end, *Braf*^*+/LSL-V600E*^*;CreER*^*+/0*^ mice (referred to as BVE mice hereafter), which spontaneously develop early-stage lung adenomas (Kamata et al., 2015), were generated on the *Stc1*^*+/+*^ and *Stc1*^*−/−*^ backgrounds. Consistent with the SPK model, significantly shorter survival of *Stc1*^*−/−*^ BVE mice was observed (Figure 2A), as well as increased lung weight (Figure 2B). Although the tumors in this model maintained histopathological features of papillary adenomas without malignant progression, accelerated stroma development was associated with reduced lung alveolar space (Figure 2C), suggesting this is the leading cause of fatal respiratory failure in these mice (Figure 2A).

Flow cytometry quantitation showed that the SPC^+^ cell number was not significantly increased in the *Stc1*^*−/−*^ compared with the *Stc1*^*+/+*^ BVE lung (Figures 2D and 2E), but significant increases of CD45^+^ and CD45^−^SPC^−^ cells were observed (Figure 2D). Importantly, these stromal cell populations did not show clear recombination of the *Braf*^*LSL-V600E*^ allele (Figure S1E), indicating that their expansion was not due to aberrant expression of oncogenic BRAF. Although CD11b^low^CD11c^+^ cell accumulation is a characteristic of the BVE model (Figure S2A) (Kamata et al., 2015), the number of these cells was not significantly increased in the *Stc1*^*−/−*^ BVE lung when adjusted for lung weight (Figure 2E). CD11c^+^ cells in the *Stc1*^*+/+*^ BVE lung were mostly negative for F4/80 (Figure 2F) but showed a cell-surface marker profile comparable to those in the SPK lungs (Figure S2A), indicating that they are not DCs but belong to the macrophage-lineage as reported (Kamata et al., 2015). Interestingly, in the *Stc1*^*−/−*^ BVE lung, CD11c^+^F4/80^+^ cells equivalent to TAMs in the SPK lungs were significantly increased, even after adjustment for lung weight (Figure 2E) and comprised up to 20%∼40% of the CD11c^+^ cells (Figure 2F). Because TAMs are derived from circulating precursors that mature into TAMs in the TME (Franklin et al., 2014, Movahedi et al., 2010, Tymoszuk et al., 2014), this increase likely reflects enhanced on-site differentiation of F4/80^−^ IMCs toward F4/80^+^ TAMs, although we cannot formally exclude the possibility that they are derived from tissue-resident macrophages, as reported in pancreatic cancer models (Zhu et al., 2017). The CD11c^+^ cells in the *Stc1*^*−/−*^ BVE lung also showed decreased MHCII and increased CCR7 (Figure S2B), which are also indicators of IMC maturation toward AM-like and pro-inflammatory macrophages, respectively (Aran et al., 2019, Jablonski et al., 2015).

For CD45^−^ populations, significant increases of CD45^−^CD31^+^Sca1^+^ endothelial and CD45^−^CD31^−^Sca1^+^ mesenchymal progenitor (Summer et al., 2007) cells were observed in the *Stc1*^*−/−*^ BVE lung (Figure 2E). Furthermore, in a similar way to the KRAS model, αSMA^+^ cells were more abundant in the stroma of the *Stc1*^*−/−*^ BVE lungs (Figure 2G). Collectively, these data show that accumulation of CD11c^+^F4/80^+^ TAMs, CD45^−^CD31^+^Sca1^+^ endothelial cells, and αSMA^+^ TAFs is a common phenotype induced by *Stc1* deficiency in the SPK/BVE models.

### STC1 Is Secreted from TAFs and Distributes to Extracellular Spaces

**Figure 3:**
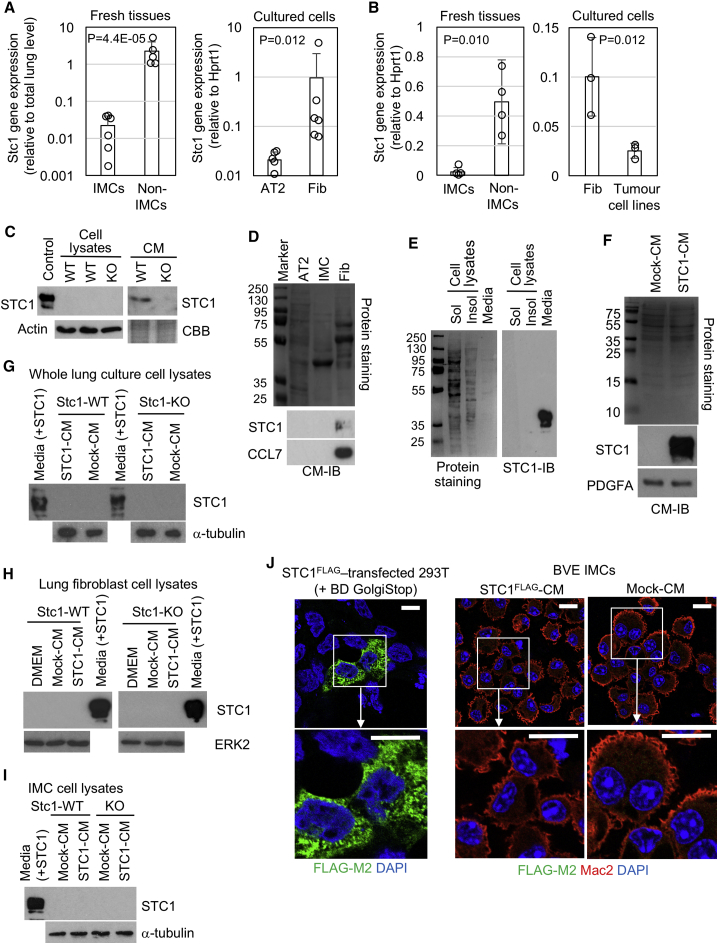
STC1 Is Expressed and Secreted by TAFs (A) *Stc1* mRNA expression in fresh IMC/non-IMC populations from *Stc1*^*+/+*^ BVE lungs (left) and cultured AT2 (tumor cell-rich)/fibroblast populations (right), as determined by qRT-PCR. *Stc1* expression is relative to total lung expression levels (left) or normalized to *Hprt1* (right). (B) *Stc1* mRNA expression in freshly isolated IMC/macrophage and non-IMC populations (left), and culture-enriched lung fibroblasts and established tumor cell lines (right), from *Stc1*^*+/−*^ SPK lungs. *Stc1* expression determined by qRT-PCR was normalized to *Hprt1*. (C) STC1 protein expression in *Stc1*^*+/+*^ (WT) fibroblasts enriched in culture from the BVE lung. STC1 protein levels in whole-cell lysates and in CM (10 times concentrated) were determined by immunoblotting. Recombinant STC1 (Control) and *Stc1*^*−/−*^ (KO) fibroblast samples served as positive and negative controls, respectively. Actin immunoblotting and Coomassie brilliant blue (CBB) staining served as loading controls. (D) STC1 immunoblotting of concentrated CM from short-term cultures of *Stc1*^*+/+*^ AT2, IMC, and fibroblast (Fib) populations. Total protein staining (top, amido-black) and immunoblots for STC1 and CCL7 (bottom) are indicated. (E) STC1 immunoblotting of NP40-soluble (Sol)/insoluble (Insol) lysates and culture media from *STC1*-transfected HEK293^T^ cells; 5% of each sample was loaded to estimate the relative STC1 distribution in each compartment. Total protein staining (left, amido-black) shows protein quantity loaded for each sample. (F) STC1 immunoblotting of CM from mock and STC1-transfected HEK293^T^ cells. Total protein staining (amido-black) and platelet-derived growth factor subunit A (PDGFA) immunoblot served as loading controls. (G–I) STC1 immunoblotting of whole-cell lysates from Stc1-WT/KO BVE total lung (G), lung fibroblast (H), and IMC (I) cultures treated with mock- or STC1-CM for 2 h. STC1-CM (media) was loaded as a positive control. α-Tubulin/ERK2 blots served as loading controls. (J) Confocal imaging of exogenous, FLAG-tagged STC1 (STC1^FLAG^) taken up by IMCs. IMCs were treated with CM from mock or STC1^FLAG^ transfected HEK293^T^ cells for 2 h and immunostained with FLAG-M2 antibody, together with Mac2, to track exogenous STC1 uptake by IMCs. STC1^FLAG^-transfected HEK293^T^ cells treated with BD GolgiStop served as positive controls for FLAG-M2. Scale bars, 10 μm.

To identify the cell population(s) expressing STC1, we undertook cell fractionation experiments, as previously described for the BVE lung (Kamata et al., 2015). *Stc1* mRNA expression was found to be ∼100 times greater in cells depleted for IMCs (the non-IMC fraction) than in IMCs (Figure 3A). When the non-IMC fraction was enriched for either AT2/tumor cells or fibroblasts by short-term culture, *Stc1* gene expression was predominantly detected in the fibroblast cultures (Figure 3A). Cultured fibroblasts showed myofibroblast characteristics with αSMA/vimentin expression and were not accompanied by recombination of the *Braf*^*LSL-V600E*^ allele (Figure S3).

For the SPK lung tissue, we purified CD11c^+^ IMC/TAMs using the same method as the BVE lung (Figure S4) and found ∼20 times higher *Stc1* mRNA expression in the IMC/TAM-depleted (non-IMC/TAM) population than in IMC/TAMs (Figure 3B). Within a week of culture, the non-IMC/TAM population developed ^G12D^KRAS-expressing tumor cell islands with confluent fibroblasts, from which lung fibroblasts were propagated by serial passage (Figure S4C). After optimization of our protocol, we were able to establish three independent tumor cell lines from the *Stc1*^*+/−*^ SPK lung (Figure S4C; Table S1), whereas attempts to generate tumor cell lines from the *Stc1*^*+/+*^ SPK lung were not successful (Table S1). However, the *Stc1*^*+/−*^ SPK tumor cell lines expressed much lower levels of *Stc1* mRNA than *Stc1*^*+/−*^ SPK fibroblasts (Figure 3B), demonstrating that, as with the BVE model, *Stc1* is predominantly expressed from lung fibroblasts in the SPK model.

Although previous studies have proposed that STC1 distributes to the mitochondria (Wang et al., 2009), we did not detect intracellular STC1 in fibroblasts derived from the *Stc1*^*+/+*^ BVE lung (Figure 3C). In contrast, STC1 was detected in conditioned media (CM) along with CC chemokine 7 (CCL7), but was not detected in CM derived from *Stc1*^*+/+*^ BVE AT2 cells or IMCs (Figures 3C and 3D). These data are consistent with a previous study reporting CCL7 and STC1 as secretory proteins upregulated in TAFs (Rajaram et al., 2013).

Extracellular STC1 has been suggested to function as a hormone that can be endocytosed by target cells (Wang et al., 2009). To investigate STC1 endocytosis, we first obtained CM from HEK293^T^ cells ectopically expressing STC1 protein. Consistent with the fibroblast data, virtually all STC1 was detected in the CM (Figure 3E) and mock-transfected HEK293^T^ cells did not secrete endogenous STC1 (Figure 3F). We then incubated primary whole lung, lung fibroblast, and IMC cultures with STC1-transfected HEK293^T^ CM (Figures 3G–3J). However, no intracellular STC1 was detected by either immunoblotting or immunofluorescence, suggesting that extracellular STC1 is not readily endocytosed by BVE lung cells. Overall, our data demonstrate that STC1 is expressed and secreted by TAFs into the mouse lung TME and that secreted STC1 predominantly distributes to extracellular spaces with no evidence for endocytosis by target cells.

### Extracellular STC1 Interacts with STC2 and GRP94

**Figure 4:**
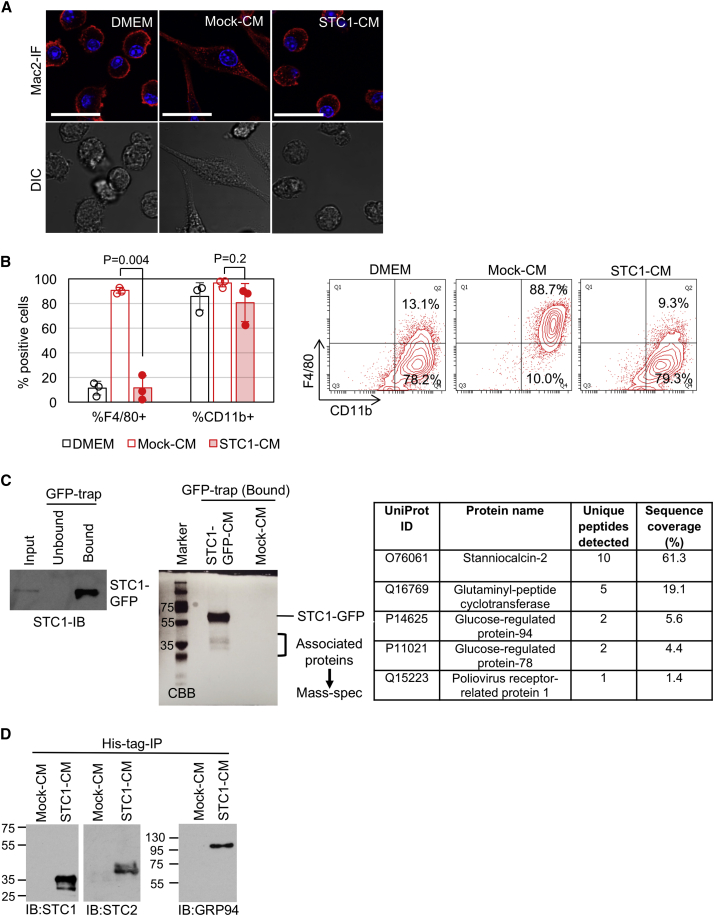
Extracellular STC1 Inhibits IMC Differentiation (A) Confocal microscopy (Mac2-IF) and differential interference contrast (DIC) imaging of *STC1*^*+/+*^ IMCs treated with 50% mock- or STC1-CM for 5 days in the presence of 5% fetal bovine serum (FBS). DMEM culture served as a negative control. Scale bars, 20 μm. (B) *STC1*^*+/+*^ IMCs treated as in (A) were analyzed for F4/80 and CD11b expression by flow cytometry. The left bar graph shows F4/80^+^ and CD11b^+^ percentages (n = 3). Representative flow cytometry plots are shown on the right. (C) Identification of STC1-binding secretory proteins. STC1-GFP protein secreted by the transfected HEK293^T^ cells was immunoprecipitated using GFP-trap (left, “bound” fraction) and resolved on SDS-PAGE to visualize STC1-associated proteins by CBB staining (middle). Five candidate proteins with signal peptides were identified by mass spectrometry analysis of the excised protein bands (right). (D) Co-immunoprecipitation of secreted endogenous STC2 or GRP94 with His-tagged STC1. CM of HEK293^T^ cells transfected with His-tagged STC1 (STC1-CM) were immunoprecipitated for the His-tag (His-tag-IP), followed by STC1, STC2 or GRP94 immunoblotting.

A consistent phenotype in the SPK/BVE models after *Stc1* deletion is accumulation of F4/80^+^ TAMs in the TME. To gain a handle on the mechanisms involved, we incubated *Stc1*^*+/+*^ BVE-derived IMCs with CM from mock or STC1-transfected HEK293^T^ cells. In the presence of the HEK293^T^ CM, the IMCs differentiated into Mac2^+^ spindle-shaped macrophages with high levels of F4/80 expression (Figures 4A and 4B). However, the presence of STC1 resulted in significant suppression of IMC differentiation and lowlevels of F4/80 expression (Figures 4A and 4B). Because extracellular STC1 is not taken up by IMCs at detectable levels (Figure 3), we reasoned that this is most likely attributable to extracellular STC1 interfering with macrophage differentiation factors within the HEK293^T^ CM.

To search for macrophage differentiation factors interacting with STC1, we immunoprecipitated an STC1-GFP fusion protein from CM of transfected HEK293^T^ cells. Co-immunoprecipitated proteins were then identified by mass spectrometry (Figure 4C). Five interacting proteins with signal sequences were identified; among which, STC2, a paralog of STC1 (Chang and Reddel, 1998), had the highest peptide sequence coverage (Figure 4C). The STC1/2 interaction was subsequently confirmed by immunoblot analysis (Figure 4D). In addition, the endoplasmic reticulum (ER)-chaperones 78 kDa/94 kDa glucose-regulated proteins (GRP78/GRP94) (Lee, 2014) were identified (Figure 4C), and the interaction of GRP94 with STC1 was confirmed by immunoblot analysis (Figure 4D).

### STC1 Prevents TAM Differentiation by Sequestering GRP94 Interaction with Scavenger Receptors

**Figure 5:**
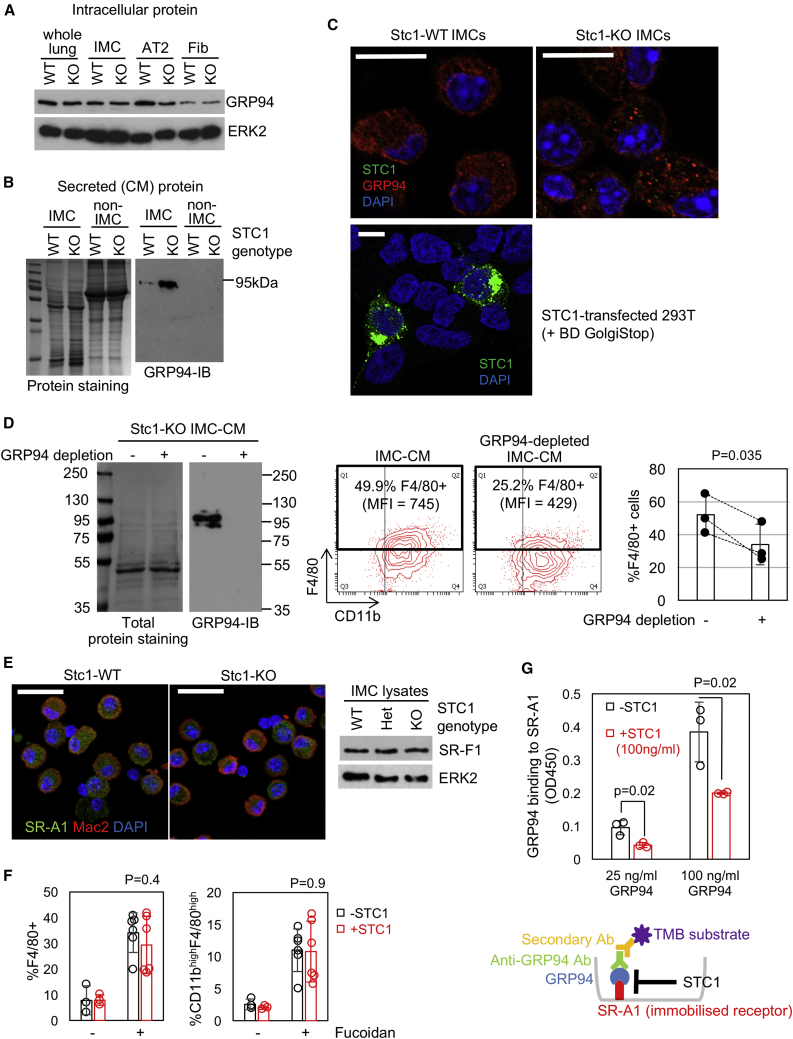
Extracellular STC1 Interferes with GRP94 (A) Intracellular GRP94 protein expression in primary cultures of fractionated IMCs, AT2, and fibroblasts (Fib) populations from Stc1-WT/KO BVE mice, as determined by immunoblotting. ERK2 blots served as loading controls. (B) Immunoblot detection of GRP94 secreted into CM during 3 days of culture of IMC and non-IMC populations from Stc1-WT/KO BVE mice. Total protein (CBB) staining served as the loading control. (C) Confocal imaging of Stc1-WT/KO IMCs for GRP94/STC1 immunofluorescence. STC1-transfected HEK293^T^ cells treated with BD GolgiStop served as positive controls for STC1 staining. Scale bars, 10 μm. (D) *Stc1*^*+/+*^ IMCs cultured for 72 h in IMC-CM depleted for GRP94 were evaluated by flow cytometry for F4/80 induction. The efficacy of immunodepletion was confirmed by GRP94 immunoblotting on the left. Representative F4/80 plots are indicated in the middle along with %F4/80^+^ and F4/80 MFI. The bar graph in the right shows %F4/80 reduction by GRP94 depletion (n = 3). (E) Scavenger receptor expression in IMCs. Confocal imaging of Stc1-WT/KO IMCs stained for SR-A1 and Mac2 (left). Maximum intensity *Z* projection images are indicated. Scale bars, 20 μm. SR-F1 immunoblotting of Stc1-WT/Het/KO IMC lysates (right). ERK2 blots served as loading controls. (F) Fucoidan-induced IMC maturation in the presence of STC1. %F4/80^+^ cells (left) and %CD11b^high^F4/80^high^ cells (right) were measured after 48 h of incubation ± fucoidan in 50% STC1-CM. (G) ELISA assay quantitation of recombinant GRP94 binding to immobilized SR-A1 in the presence of 100 ng/mL recombinant STC1 (n = 3).

We were particularly interested in the interaction of STC1 with GRP94 because it is well-established that extracellular GRP94 regulates innate and adaptive immunity (Binder et al., 2000, Srivastava, 2002, Yang et al., 2007, Zheng et al., 2001). We investigated the expression of GRP94 and found that, although intracellular GRP94 was expressed in different cell types within the BVE lung (Figure 5A), secreted GRP94 was only detectable in IMC-CM and was elevated in CM derived from *Stc1*^*−/−*^ IMCs (Figure 5B). Endogenous intracellular STC1 was barely detectable in *Stc1*^*+/+*^ IMCs (Figures 3I and 5C), suggesting that the relatively low GRP94 secretion by *Stc1*^*+/+*^ IMCs is not attributable to intracellular interactions between STC1 and GRP94 proteins but may reflect the phenotypic differences between *Stc1*^*+/+*^ and *Stc1*^*−/−*^ IMCs (Figures 2F and S2B).

Primary IMCs are difficult to manipulate genetically, and therefore, we used immunodepletion approaches to downregulate GRP94 from IMC-CM (Figure 5D). We found that differentiation of IMCs was significantly less prominent after culture with GRP94-deplected CM. This suggests that endogenous GRP94 secreted by IMCs functions as an autocrine macrophage differentiation factor. The same trend was observed when HEK293^T^-CM was used for GRP94 depletion, although the inhibitory effects on IMC differentiation were more modest (Figures S5A and S5B).

Macrophages are known to express two related GRP94 receptors: scavenger-receptors class-A member-1 (SR-A1) and class-F member-1 (SR-F1) (Berwin et al., 2004, Berwin et al., 2003), and expression of these receptors in BVE-derived IMCs was confirmed by immunofluorescence/immunoblotting (Figure 5E). GRP94 association with SR-A1 was also detected on the cell surface of IMCs by confocal imaging (Figures S5C and S5D). Fucoidan is a sulphated polysaccharide that engages with SR-A1 (Hsu et al., 1998), and we found that fucoidan treatment of IMCs induced their TAM differentiation (Figure S5E). This was not disrupted by exogenous STC1 (Figure 5F), suggesting that STC1 does not interfere with activation of scavenger receptors *per se*. We, therefore, reasoned that the most likely mode of action for exogenous STC1 is through the sequestering of GRP94 from engagement with scavenger receptors. To investigate this more directly, we performed *in vitro* binding assays using ELISA detection. Binding of GRP94 to immobilized SR-A1 was substantially attenuated in the presence of recombinant STC1 (Figure 5G), and there was a modest, but significant, reduction of exogenous GRP94 uptake by cultured IMCs in the presence of STC1 (Figure S5F).

To further elucidate the functional roles for SR-A1 in IMCs, we treated IMCs with the SR-A1 inhibitor rhein (Yuan et al., 2015). Because SR-A1 mediates p38 mitogen-activated protein kinase (MAPK) and AKT phosphorylation (Jin et al., 2009), we examined their phosphorylation in IMCs cultured with rhein. As expected, rhein effectively inhibited p38 MAPK/AKT phosphorylation (Figure S6A), demonstrating the contribution of SR-A1 to autocrine activation of p38/AKT in IMCs. Furthermore, prolonged exposure to rhein induced cell death of IMCs (Figure S6B), suggesting an essential role for SR-A1 in IMC survival.

Collectively, our data suggest that GRP94 functions as an autocrine maturation factor for IMCs and that extracellular STC1 sequesters it from interacting with its cognate scavenger receptors that also support IMC survival, thus contributing to maintenance of the immature state of IMCs in the TME.

### STC1 Regulation of TAFs Is Dependent on an Interaction with TAMs

**Figure 6:**
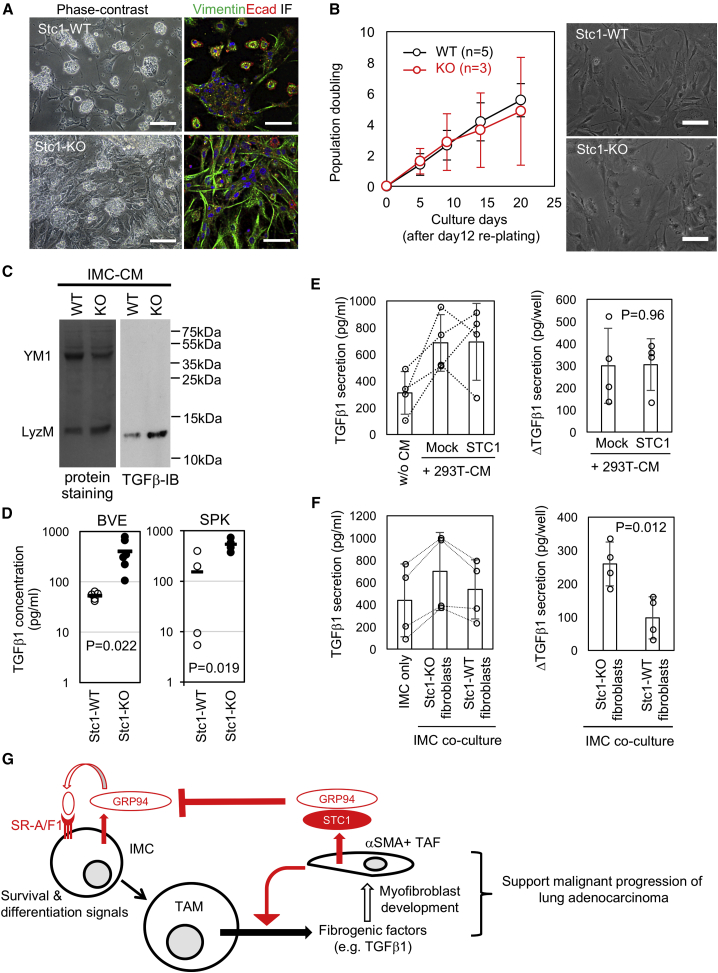
Analysis of TAFs and TGF-β Production from TAMs (A) Phase-contrast (left) and vimentin/E-cadherin confocal (right) images of primary cultures of Stc1-WT/KO BVE lung. Scale bars, 100 μm (left) or 50 μm (right). (B) *In vitro* long-term growth of fibroblasts purified from Stc1-WT/KO BVE lungs. The left line graph shows average population doublings (n = 3–5). Representative morphology on day 20 are indicated on the right (scale bars, 50 μm). (C) TGF-β immunoblotting of 72 h CM of Stc1-WT/KO BVE-derived IMC cultures. Total protein staining (left, amido-black) served as a loading control. (D) TGF-β1 ELISA of 72 h CM of Stc1-WT/KO BVE-derived (left, n = 4–6) and SPK-derived IMC/TAM (right, n = 4) cultures. (E) TGF-β1 ELISA of CM from Stc1-KO BVE-derived IMC culture treated with 50% mock and STC1-transfected HEK293^T^ CM (293T-CM) for 72 h (n = 4). The left bar graph shows raw data, whereas the right shows net increases of TGF-β1 secretion by 293^T^-CM treatment. (F) TGF-β1 ELISA of CM from Stc1-KO BVE-derived IMCs co-cultured with STC1-KO/WT lung fibroblasts for 72 h (n = 4). The left bar graph shows raw data, whereas the right shows a net increase of TGF-β1 secretion after co-culture. (G) A diagram of the proposed model showing mechanism of regulation of IMC maturation to TAMs by STC1 and consequent effect on TGF-β secretion and TAF accumulation.

The fibroblast-derived origin of STC1 (Figure 3) and TAF/myofibroblast phenotype (Figures 1G and 2G) suggests that *Stc1* deficiency affects TAFs as well as IMCs. Indeed, short-term culture of *Stc1*^*−/−*^ BVE whole lung tissue resulted in the greater accumulation of vimentin^+^ fibroblasts than *Stc1*^*+/+*^ BVE lung cultures (Figure 6A), reflecting the *in vivo* pathology. However, the growth rate of fibroblasts isolated from *Stc1*^*−/−*^ BVE lung tissue was not increased compared with *Stc1*^*+/+*^ BVE lung fibroblasts (Figure 6B). This shows that STC1 deficiency does not affect intrinsic fibroblast growth.

Instead, we focused on investigating TGF-β, because TGF-β is a well-known driver of TAF/myofibroblast development (Kalluri and Zeisberg, 2006, Wynn and Ramalingam, 2012) and IMCs are known to secrete this fibrogenic factor (Kamata et al., 2015). We found that *Stc1*^*−/−*^ IMCs secreted significantly more mature TGF-β1 than did *Stc1*^*+/+*^ IMCs by immunoblot analysis (Figure 6C). Similar results were obtained by ELISA quantitation of TGF-β1 in the CM from IMC/TAMs isolated from both BVE and SPK lungs (Figure 6D).

The above data suggest that fibroblast-derived STC1 may suppress IMC secretion of TGF-β in a paracrine manner. However, we found that TGF-β1 secretion by *Stc1*^*−/−*^ IMCs was not inhibited in the presence of exogenous STC1 (Figure 6E). When this experiment was repeated in the presence of TAFs, there was significantly enhanced secretion of TGF-β1 from IMCs co-cultured with *Stc1*-deficient TAFs compared with *Stc1*^*+/+*^ TAF co-cultures (Figure 6F). Thus, although extracellular STC1 does not directly suppress IMC secretion of TGF-β1, TAF-derived STC1 affects TGF-β1 production by IMCs in a paracrine manner, and this production is dependent on an interaction between TAFs and IMCs.

Because the SR-A1 inhibitor rhein is known to suppress pancreatic fibrosis *in vivo* (Tsang et al., 2013), we sought to determine whether rhein affects TGF-β1 secretion by IMCs and, indeed, found this to be the case (Figure S6C). Chemical inhibition of TGF-β1 signaling by SB431542 (Callahan et al., 2002) attenuated αSMA expression in TAFs co-cultured with IMCs (Figures S6D and S6E), but it did not affect their proliferation (Figure S6F), demonstrating that IMC-derived TGF-β1 promotes myofibroblast differentiation but not proliferation.

Taken together, the proposed role of STC1 in regulating TAMs and TAFs in the TME through scavenger receptors and TGF-β1 signaling is shown in Figure 6G.

### *STC1* in Human Lung Adenocarcinoma

**Figure 7:**
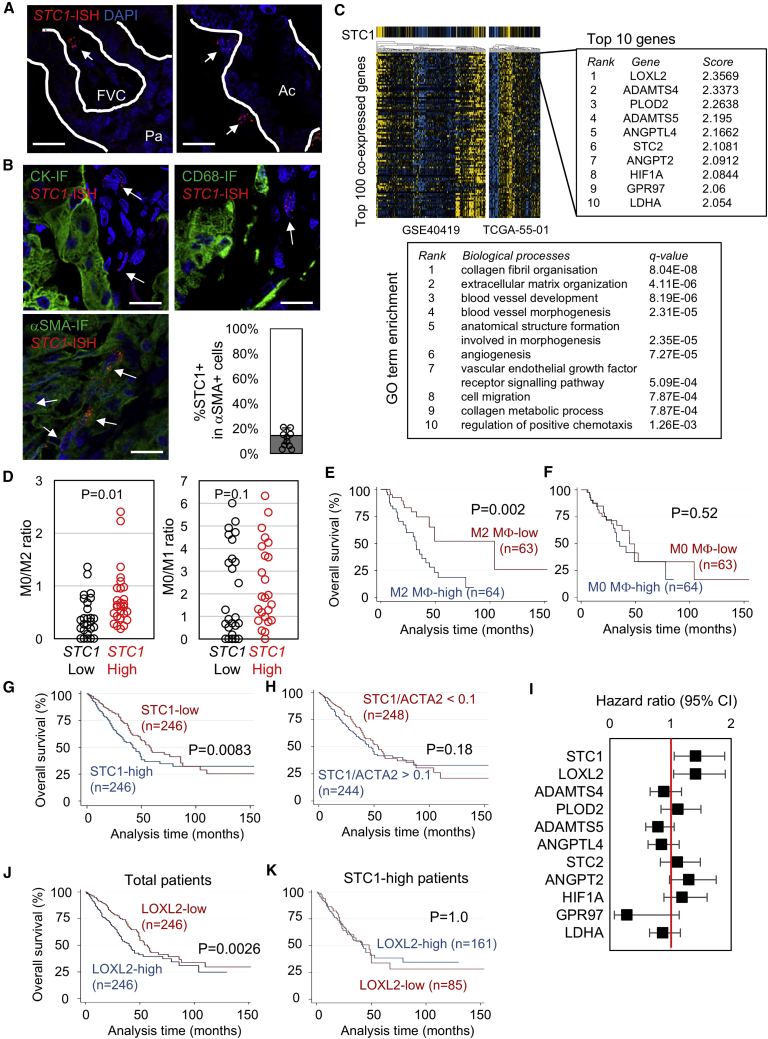
Myofibroblast *STC1* Expression and the Link with TAM Immatureness in Human Lung Adenocarcinoma (A) *STC1* mRNA detected in the FVC in papillary (Pa) lesions (left) and in the stroma in acinar (Ac) lesions (right) by ISH. Scale bars, 20 μm. (B) *STC1*-ISH combined with immunofluorescence for pan-cytokeratin (CK, top left), CD68 (top right) and αSMA (bottom left). Scale bars, 20 μm. The bar graph (bottom right) shows %STC1^+^ in αSMA^+^ cells from 10 tissue microarray cores. (C) Analysis of the genes co-expressed with *STC1* using the SEEK web interface. Clustering of two datasets (GSE40419 and TCGA-55-01) for the top 100 co-expressed genes is shown on the left with the top 10 co-expressed genes highlighted on the upper right. The lower right shows gene ontogeny (GO) term-enrichment analysis of the top 100 co-expressed genes. (D) CIBERSORT analysis of M0/M2 (left) and M0/M1 (right) ratios in *STC1*^high/low^ cases (top and bottom, 20% *STC1* mRNA expression) in the TCGA lung adenocarcinoma dataset containing 127 samples satisfying the threshold (p < 0.05) of CIBERSORT analysis. The p values are calculated with the Mann-Whitney U test. (E and F) Kaplan-Meier analysis of TCGA patients with lung adenocarcinoma for overall survival of high/low (median cutoff) M2 (E) or M0 (F) macrophage-abundance groups. (G) Kaplan-Meier analysis of overall survival of *STC1*^high/low^ (RNA sequencing [RNA-seq], above/below-median) patients from the TCGA Pan-Cancer Atlas database. (H) The same samples as in (G) were grouped according to the *STC1/ACTA2* mRNA expression ratio (above or below 0.1), and their overall survival was compared by Kaplan-Meier analysis. (I) Survival impact of the top 10 *STC1* co-expressed genes as assessed with the Kaplan-Meier Plotter. Hazard ratios (black squares) with 95% confidence intervals (error bars) were obtained for each gene by comparing above- and below-median groups. (J and K) Kaplan-Meier analysis of overall survival of *LOXL2*^high/low^ (above- and below-median) samples obtained as in (G). Total (J) and *STC1*^high^ (above-median) (K) samples were analyzed.

To investigate whether STC1 operates in a similar way in human lung adenocarcinoma, we first examined *STC1* expression by *in situ* hybridization (ISH) and detected *STC1*-expressing cells sporadically in the TME (Figure 7A). ISH/immunofluorescence (IF) dual staining revealed that *STC1*-expressing cells were negative for pan-cytokeratin and the macrophage marker CD68, but a fraction of the cells expressing the TAF marker αSMA were positive for *STC1* mRNA (mean = 14.3%; Figure 7B). In addition, analysis of transcriptome datasets of human lung adenocarcinomas (Table S2) using the web interface SEEK (Zhu et al., 2015) demonstrated that the genes co-expressed with *STC1* are enriched for regulators of ECM organization, collagen fibril biogenesis, angiogenesis, tissue morphogenesis/cell migration, and chemotaxis (Figure 7C; Table S3). Notably, collagen cross-linking enzymes *LOXL2* and *PLOD2*, which are reportedly expressed in TAFs (Torres et al., 2015), were the top-ranked genes (Figure 7C). These results are consistent with *STC1* expression being restricted to αSMA^+^ TAFs (Figure 7B).

STC1 inhibition of TAM maturation is a key finding of our animal studies (Figure 6G). Although it is unclear which type(s) of macrophage in the TME of human cancers is or are functionally equivalent to IMCs, we assumed that functional maturation of naive M0-like macrophages into M2-like TAMs may be analogous to IMC maturation in mice. Accordingly, we calculated the M0/M2 macrophage ratio in *STC1*^high^ and *STC1*^low^ cases in the Cancer Genome Atlas (TCGA) lung adenocarcinoma dataset (Cancer Genome Atlas Research, 2014) using the web-tool CIBERSORT (Newman et al., 2015) and found a significant increase in the M0/M2 ratio in *STC1*^high^ compared with *STC1*^low^ cases. Interestingly, the M0/M1 ratio was not significantly different (Figure 7D), suggesting that the effect of *STC1* on TAM maturation is restricted to M2-like TAMs.

We observed a negative prognostic effect of M2-like TAMs on survival of TCGA lung adenocarcinoma (Figure 7E), but there was no effect of “immature M0” TAMs (Figure 7F). Combining the observation of higher M0/M2 ratio in *STC1*^high^ lung adenocarcinomas (Figure 7D) and a worse prognostic outcome for patients with higher M2 TAMs (Figure 7E), the prediction from these data is that patients with *STC1*^high^ have better prognostic outcome. However, unexpectedly, we found high *STC1* expression was associated with poorer prognosis (Figure 7G). We reasoned that this contradiction may be explained by high *STC1* expression reflecting an abundance of αSMA^+^ stromal myofibroblasts, rather than being a direct biological effect of STC1 *per se*. Indeed, we did not observe a statistically significant survival effect of *STC1* expression when its expression level was normalized against *ACTA2*, the gene encoding αSMA (Figure 7H). We further extended the survival analysis to top-ranked genes co-expressed with *STC1* (Figures 7C and S7) and identified *LOXL2* as having a poor prognostic effect in a similar manner to STC1 (Figures 7I and 7J). Interestingly, the negative effect of high *LOXL2* expression was completely abrogated when the analysis was restricted to patients with *STC1*^high^ (Figure 7K). This observation is supportive of the negative prognostic effect of *LOXL2-*expressing cells being neutralized in an STC1-rich microenvironment, a result consistent with the suppressive role of STC1 as suggested by our mouse data.

## Discussion

In this report, we identified fibroblast-derived STC1 as a key paracrine modulator of the TME in lung adenocarcinoma. With GEM models, we show that STC1 secreted by lung fibroblasts inhibits TAM maturation, leading to suppression of lung adenocarcinoma development (see Figure 6G for model). By *ex vivo* studies of primary cells, we show that STC1 operates, at least in part, to suppress TAM differentiation by sequestering the binding of GRP94 from interaction with scavenger receptors. Our analysis of human lung adenocarcinomas is consistent with the myofibroblast-restricted expression of STC1 and a role in TAM modulation. Overall, this is a novel mechanism of regulation of TAM differentiation that has not previously, to our knowledge, been reported but has important implications for our understanding of TAM plasticity, which is a well-characterized factor in tumor progression.

The lineage origin of the IMCs/TAMs identified in our mouse system is not presently clear. Although TAMs are thought to mostly derive from circulating precursors in the mononuclear phagocyte lineage (Franklin et al., 2014, Movahedi et al., 2010, Tymoszuk et al., 2014), recent studies have implied a contribution of tissue-resident macrophages to TAMs in pancreatic (Zhu et al., 2017) and lung metastasis (Loyher et al., 2018) models. In the previously reported lung-metastasis model, resident IM-derived, CD11b^high^ TAMs were accumulated (Loyher et al., 2018), whereas the CD11b^low^CD11c^+^Siglec-F^+^ IMC/TAMs detected in our models resemble resident AMs rather than IMs (Misharin et al., 2013). Of note, however, IMCs accumulated in our models are largely devoid of F4/80 expression and thus are unlikely to derive directly from F4/80^+^ AMs. We speculate that the F4/80^−^ IMCs observed are a cell type similar to the monocyte-derived Siglec-F^+^ AMs (Misharin et al., 2017) or transitional macrophages (Aran et al., 2019) recently reported in lung fibrosis models. Lineage-tracing approaches will be required to clarify the origin of these cells in the future.

We found that STC1 is exclusively secreted by TAFs cultured *ex vivo* (Figure 3D), and there is almost no retention of intracellular STC1 within these cells (Figure 3C). This observation contradicts previous studies, which highlighted functions for intracellular STC1 in regulation of oxidative stress (Yeung et al., 2012). Secreted STC1 has been reported to localize to mitochondria when taken up by target cells (Wang et al., 2009), but our biochemical analysis does not support endocytosis of extracellular STC1 (Figures 3G–3I). Instead, we found that STC1 operates by interacting with other secreted proteins in the extracellular environment.

We identified GRP94 as an STC1-interacting secretory protein that promotes IMC maturation, but the mechanistic basis for this process remains unclear. Engagement of the GRP94 receptor SR-A1 has been reported to activate signaling pathways, including phospholipase C, PI3 kinase, and protein kinase C (Hsu et al., 1998, Jin et al., 2009), which have vital roles in macrophage differentiation and survival (Stanley and Chitu, 2014). Consistently, GRP94 immunodepletion suppressed IMC differentiation by 20%–30% (Figure 5D), and chemical inhibition of SR-A1 for a prolonged time induced cell death of cultured IMCs (Figure S6B). These data suggest that GRP94, together with other as-yet unidentified autocrine factors, contributes to IMC differentiation. GRP94/SR-A1-mediated IMC survival may be a prerequisite for maturation induction by other autocrine factors.

Apart from GRP94, four candidate STC1-interacting secretory proteins were identified: STC2, glutaminyl-peptide cyclotransferase (QPCT), GRP78, and poliovirus receptor-related protein 1 (PVRP1) (Figure 4C). Although STC1 forms homodimers through the C-terminal cysteine (Trindade et al., 2009), it has been unclear as to whether STC1 forms heterodimers with STC2 because the C-terminal cysteine is not spatially conserved in STC2 (Ishibashi and Imai, 2002). To our knowledge, this study is the first report showing STC1/STC2 heterodimerization. In human lung adenocarcinomas, *STC1* gene expression is well correlated with *STC2* (Figure 7C), suggesting that STC1/2 is expressed by similar cell types. GRP78 has been shown to exert immunomodulatory functions (Corrigall et al., 2004) and contribute to the pathogenesis of lung fibrosis (Ayaub et al., 2016) and macrophage maturation (Kim et al., 2018). QPCT is a secretory enzyme that catalyzes cyclization of N-terminal glutamine to stabilize CC chemokines CCL2 and CCL7 (Cynis et al., 2011). PVRP1 is a member of the immunoglobulin superfamily of cell-adhesion molecules (Takai et al., 2008), and its soluble secreted isoform has been also identified (Lopez et al., 2001). PVRP1 is also known as a cell-surface receptor for herpes simplex virus (Geraghty et al., 1998), suggesting that PVRP1 may function as a cell-surface receptor for STC1. In the future, it will be interesting to further examine the roles of these novel STC1 interactions in TAM maturation and tumor development.

Although TAF accumulation is a characteristic of *Stc1* deficiency in our mouse models, we observed no differences in the growth rates of *Stc1*^*−/−*^ TAFs after *ex vivo* culture (Figure 6B). Therefore, we attribute the *Stc1*^*−/−*^ TAF phenotype to increased secretion of TGF-β1 from TAMs. Such a paracrine function of STC1 is analogous to the previously documented paracrine function of mesenchymal stem cell (MSC)-derived STC1 that inhibits lung fibrosis by suppressing TGF-β1 production (Ono et al., 2015). Given the similarity between MSCs and TAFs with activated fibroblast phenotypes (Kalluri, 2016), STC1-producing MSCs/TAFs may share similar anti-fibrotic functions. In human lung adenocarcinomas, we found that *STC1* is expressed in only a few αSMA^+^ cells (Figure 7B), suggesting it may be a specific sub-population of phenotypically and functionally heterogeneous TAFs (Kalluri, 2016). Further studies will be needed to clarify the developmental relationship between *STC1*-expressing TAFs and MSCs.

Our data highlight future potential therapeutic options targeting TAF/TAM interactions in lung adenocarcinoma. Several inhaled peptide-based biotherapies are currently under pre-clinical or clinical development (Bodier-Montagutelli et al., 2018, Fellner et al., 2016), and animal studies for inhaled immunotherapy targeting the TME have been reported (Le Noci et al., 2016, Le Noci et al., 2015). Because intra-tracheal delivery of STC1 protein into a mouse lung fibrosis model has been shown to improve the pathology (Ono et al., 2015), it would be worthwhile to test a similar strategy for treating lung adenocarcinoma associated with the desmoplastic stroma. Our *Stc1*^*−/−*^ SPK model would serve as a pre-clinical platform for testing potentially anti-fibrogenic biomaterials, including STC1 itself, by topical delivery to the lung.

## STAR★Methods

### Key Resources Table

**Table:** 

REAGENT or RESOURCE	SOURCE	IDENTIFIER
**Antibodies**		

Biotin anti-mouse CD11b (M1/70)	Tonbo Biosciences	Cat# 30-0112, RRID;AB_2621639
PE anti-mouse Gr1 (RB6-8C5)	SouthernBiotech	Cat# 1900-09L, RRID:AB_2795466
FITC anti-mouse CD11c (N418)	BioLegend	Cat# 117305, RRID:AB_313774
APC anti-mouse CD11c (N418)	BioLegend	Cat# 117309, RRID:AB_313778
PE anti-mouse F4/80 (BM8)	BioLegend	Cat# 123109, RRID:AB_893498
PE anti-mouse CD45 (30-F11)	BioLegend	Cat# 103106, RRID:AB_312971
FITC anti-mouse CD4 (GK1.5)	BioLegend	Cat# 100405, RRID:AB_312690
PE anti-mouse CD8a (53-6.7)	BioLegend	Cat# 100708, RRID:AB_312747
Biotin anti-mouse B220 (RA3-6B2)	BioLegend	Cat# 103203, RRID:AB_312988
AF488 anti-mouse CD31 (MEC13.3)	BioLegend	Cat# 102514, RRID:AB_2161031
APC anti-mouse Sca1 (D7)	Miltenyi Biotec	Cat# 130-093-223, RRID:AB_1036101
PE anti-mouse CCR7 (4B12)	Thermo Fisher Scientific	Cat# 12-1971-80, RRID:AB_465904
PE anti-mouse Siglec F (1RNM44N)	Thermo Fisher Scientific	Cat# 14-1702-80, RRID:AB_2572865
PE anti-mouse CD103 (2E7)	BioLegend	Cat# 121405, RRID:AB_535948
PE anti-mouse CD117 (2B8)	BioLegend	Cat# 105807, RRID:AB_313216
PE anti-mouse I-A/I-E (M5/114.15.2)	BioLegend	Cat# 107607, RRID:AB_313322
FITC anti-mouse CD326 (G8.8)	BioLegend	Cat# 118207, RRID:AB_ 1134106
Rabbit anti-SP-C (FL-197)	Santa Cruz	Cat# sc-13979, RRID:AB_2185502
Mouse anti-E-Cadherin	BD Biosciences	Cat# 610182, RRID:AB_397581
Rabbit anti-Actin, smooth muscle	Abcam	Cat# ab5694, RRID:AB_2223021
Rat anti-MAC2 (M3/38)	Cedarlane	Cat# CL8942AP, RRID:AB_10060357
Rabbit anti-mouse CD204/MSR1	Sino Biological	Cat# 50129-R004
Mouse anti-Vimentin (LN-6)	Sigma-Aldrich	Cat# V2258, RRID:AB_261856
Rat anti-GRP94 (9G10)	Enzo Life Sciences	Cat# ADI-SPA-850, RRID:AB_10615091
Rabbit anti-STC1	Abcam	Cat# ab83065, RRID:AB_1861344
Rabbit anti-STC2	Bethyl Laboratories	Cat# A302-369A, RRID:AB_1907252
Rabbit anti-TGFβ (56E4)	Cell Signaling	Cat# 3709, RRID:AB_2063357
Biotin anti-mouse MCP3	Abcam	Cat# ab83427, RRID:AB_1859633
Rabbit anti-PDGF-AA	Millipore	Cat# 07-1436, RRID:AB_1587372
Mouse anti-alpha-Tubulin (B-5-1-2)	Sigma-Aldrich	Cat# T6074, RRID:AB_477582
Mouse anti-ERK2 (D-2)	Santa Cruz	Cat# sc-1647, RRID:AB_627547
Rabbit anti-phospho-p38 MAPK	Cell Signaling	Cat# 9211, RRID:AB_331641
Rabbit anti-p38 MAPK	Cell Signaling	Cat# 9212, RRID:AB_330713
Rabbit anti-phospho-Akt (Ser473) (D9E)	Cell Signaling	Cat# 4060, RRID:AB_2315049
Rabbit anti-Akt (pan) (C67E7)	Cell Signaling	Cat# 4691, RRID:AB_915783
Rabbit anti-SCARF1	Proteintech	Cat# 13702-1-AP, RRID:AB_2182983
Rabbit anti-phospho-SMAD3 (Ser423/425) (EP823Y)	Abcam	Cat# ab52903, RRID:AB_882596
Mouse anti-GAPDH (GA1R)	Thermo Fisher Scientific	Cat# MA5-15738, RRID:AB_10977387
Rat anti-mouse F4/80 (CI:A3-1)	Bio-Rad	Cat# MCA497R, RRID:AB_323279
Biotin anti-mouse IgM (II/41)	Thermo Fisher Scientific	Cat# 13-5790-82, RRID:AB_466675
Mouse anti-6x-His Tag (HIS.H8)	Thermo Fisher Scientific	Cat# MA1-21315, RRID:AB_2536982
Mouse anti-FLAG® M2	Sigma-Aldrich	Cat# F1804, RRID:AB_262044
Mouse anti-pan Cytokeratin (AE1/AE3)	Abcam	Cat# ab27988, RRID:AB_777047
Mouse anti-CD68 (PG-M1)	Agilent (DAKO)	Cat# M0876, RRID:AB_2074844

**Bacterial and Virus Strains**		

Ad5-mSPC-Cre	University of IOWA Viral Vector Core Facility	N/A

**Biological Samples**		

FFPE human lung adenocarcinoma samples	University Hospitals of Leicester NHS Trust, Pathology Department	LREC 14/EM/1159 (ethical approval held by Dr John Le Quesne)

**Chemicals, Peptides, and Recombinant Proteins**		

GIBCO Collagenase, Type I	Thermo Fisher Scientific	Cat# 17018-029
DNase I	Sigma-Aldrich	Cat# DN25
Fucoidan	Santa Cruz	Cat# sc-255187
Rhein	Sigma-Aldrich	Cat# 275611
PEI, branched 25kD	Sigma-Aldrich	Cat# 408727
Recombinant human SR-A1 protein	Bio-Techne	Cat# 2708-MS-050
Recombinant human GRP94 protein	RayBiotech	Cat# 228-21002-2
Recombinant human STC1 protein	ProSpec	Cat# HOR-259
Recombinant mouse TGFβ1 protein	Cell Signaling	Cat# 5231LC
SB431542	Cambridge Bioscience	Cat# CAY13031
eBioscience Fixable Viability Dye, eFluor™ 780	Thermo Fisher Scientific	Cat# 65-0865-14

**Critical Commercial Assays**		

Human/Mouse TGFβ1 ELISA	Thermo Fisher Scientific	Cat# 88-8350-22, RRID:AB_2575209
GenElute Mammalian Total RNA Miniprep Kit	Sigma-Aldrich	Cat# RTN350
SuperScript III Reverse Transcriptase	Thermo Fisher Scientific	Cat# 18080-093
DNA-*free* DNA Removal Kit	Thermo Fisher Scientific	Cat# AM1906
SensiFAST SYBR® No-ROX Kit	Bioline	Cat# BIO-98020
Dynabeads Protein G	Thermo Fisher Scientific	Cat# 10003D
GFP-Trap® Magnetic Agarose	ChromoTek	Cat# gtma-20, RRID:AB_2631358
ImmPRESS HRP Anti-Rat IgG polymer	Vector Laboratories	Cat# MP-7404, RRID:AB_2336531
DAB Peroxidase Substrate Kit	Vector Laboratories	Cat# SK-4100
eBioscience ELISA/ELISPOT Diluent	Thermo Fisher Scientific	Cat# 00-4202-56
eBioscience TMB Solution (1X)	Thermo Fisher Scientific	Cat# 00-4201-56
BD Cytofix/Cytoperm Fixation/Permeabilization Solution	BD Biosciences	Cat# 554714
FITC BrdU Flow Kit	BD Biosciences	Cat# 559619, RRID:AB_2617060
Novolink Polymer Detection System	Leica Biosystems	Cat# RE7140-K
SignalStain® Boost IHC Detection reagent (HRP, mouse)	Cell Signaling	Cat# 8125, RRID:AB_10547893
Opal 4-Color Manual IHC Kit	PerkinElmer	Cat# NEL810001KT
RNAscope® Probe-Hs-STC1	Bio-Techne (ACD)	Cat# 472691
RNAscope® Multiplex Fluorescent Reagent Kit v2	Bio-Techne (ACD)	Cat# 323120

**Deposited Data**		

TCGA/GEO lung adenocarcinoma datasets (used for Figure 7C)	See Table S2	See Table S2
cBioPortal – Lung Adenocarcinoma (TCGA, Nature 2014) dataset (for CIBERSORT analysis in Figures 7D and S7E)	Cancer Genome Atlas Research, 2014	http://www.cbioportal.org/study/summary?id=luad_tcga_pub
cBioPortal – Lung Adenocarcinoma (TCGA, PanCancer Atlas) dataset (for Figure S7)	TCGA	http://www.cbioportal.org/study/summary?id=luad_tcga_pub

**Experimental Models: Cell Lines**		

Human embryonic kidney HEK293^T^	ATCC	Cat# ATCC CRL-3216, RRID:CVCL_0063

**Experimental Models: Organisms/Strains**		

Mouse: B6.129S4-Kras^tm4Tyj^	Jackson et al., 2001	RRID:MGI:5440073
Mouse: B6.129P2-Braf ^tm1Cpri^	Mercer et al., 2005	RRID:MGI:3843303
Mouse: B6.Cg-Stc1^tm1Rred^	Chang et al., 2005	RRID:MGI:3626185
Mouse: B6.Cg-Tg(CAG-cre/Esr1^∗^)5Amc	Hayashi and McMahon, 2002	RRID:MGI:3845073
Mouse: B6.Cg-Kras^tm4Tyj^; Stc1^tm1Rred^	In house	N/A
Mouse: B6.Cg-Tg(CAG-cre/Esr1^∗^)5Amc; Braf ^tm1Cpri^; Stc1^tm1Rred^	In house	N/A

**Oligonucleotides**		

PCR primers	See Table S4	N/A

**Recombinant DNA**		

pLEICS29-hSTC1-TEV-EGFP	This study	N/A
pLEICS49-hSTC1-TEV-His4/FLAG3	This study	N/A
pcDNA3-hSTC1-TEV-His10	This study	N/A

**Software and Algorithms**		

ImageJ	NIH	RRID:SCR_003070
Mascot (version 2.2.04)	Matrix Science Ltd	RRID:SCR_014322
Huygens Essential	Scientific Volume Imaging	RRID:SCR_014237
Stata Statistical Software: Release 16	StataCorp LLC	RRID:SCR_012763
SEEK	Zhu et al., 2015	http://seek.princeton.edu/
Kaplan-Meier Plotter	Gyorffy et al., 2013	https://kmplot.com/
CIBERSORT	Newman et al., 2015	https://cibersort.stanford.edu/

**Other**		

FV1000 confocal laser scanning system	Olympus	N/A
LTQ-Orbitrap-Velos mass spectrometer	Thermo Fisher Scientific	N/A

### Resource Availability

#### Lead Contact

Further information and requests for resources and reagents should be directed to and will be fulfilled by the Lead Contact, Catrin Pritchard (cap8@le.ac.uk).

#### Materials Availability

Mouse lung cell lines and STC1 plasmids were newly generated in this study and are freely available upon request to the lead contact. All other reagents are not unique to this study,

#### Data and Code Availability

This study did not generate new datasets/code. TCGA lung adenocarcinoma datasets analyzed in this study were accessed through cBioPortal (http://www.cbioportal.org/). CIBERSORT was accessed at https://cibersort.stanford.edu/

### Experimental Model and Subject Details

#### Animals

All animal experiments were performed under UK Home Office License authority. *Kras*^*LSL-G12D*^ (Jackson et al., 2001), *Braf*^*LSL-V600E*^ (Mercer et al., 2005), and *CAGG-CreER*^*TM*^ (also known as Tg(CAG-cre/Esr1^∗^)5Amc) (Hayashi and McMahon, 2002) alleles were genotyped as described (Andreadi et al., 2012, Kamata et al., 2015), using primers described in Table S4. Genotyping of *Stc1* alleles (Chang et al., 2005) was performed using the following primers: 5′-AAAAGCCAGAGGTGCAAGAA-3′ and 5′-TGTGATCGGAATTCCTCGAC-3′ for the *Stc1*-targeted allele, and 5′-AGCGCACGAGGCGGAACAAA-3′ and 5′-AGAGAGCCGCTGTGAGGCGT-3′ for the *Stc1* wild-type allele. All experimental animals were maintained on a C57BL/6J background. 5-10 week-old BVE mice with random sex distribution were used for survival studies, while age/gender-matched BVE mice were used for lung weight and tissue analyses (51 days of age in average for weight analysis, and 6 weeks of age for tissue analysis, respectively). Nasal delivery of 1x10^8^ pfu Ad5-mSPC-Cre adenovirus to 10-15 week-old *Kras*^*LSL-G12D*^ mice with random sex distribution was performed as described (Kamata et al., 2015), to produce experimental SPK mice. SPK mice at 130 – 510 days post induction were used for survival studies, while age/gender-matched SPK mice were used for lung weight and tissue analyses (330 days post induction in average for weight analysis, and 9 months post induction for tissue analysis, respectively). Lung tissues were processed as described (Kamata et al., 2015) for H&E and immunohistochemistry.

#### Primary mouse lung tumor cell lines

To establish mouse lung tumor cell lines from SPK lung tumor tissues, the lung tissues depleted for macrophage-lineage cells (see below, METHOD DETAILS, Cell purification) were cultured in Dulbecco’s modified Eagle medium containing 10% FBS (DMEM/10% FBS) for 3 days. On day 3, floating dead cell and non-adherent hematopoietic cells were removed, and live adherent cells were fed with serum-free DMEM (to avoid fibroblast outgrowth) and cultured for 2 weeks without passage. Then, the cultures were maintained in DMEM/2% FBS without passage until immortalized cells were observed at 4-9 weeks. Once immortalized, the cells were trypsinised and propagated in DMEM/5% FBS. The tumor cell origin of the immortalized cell lines was confirmed by *Kras* recombination PCR as described (Kamata et al., 2017) (Table S4). One cell line from *Stc1*^*−/−*^ and three lines from *Stc1*^*+/−*^ tumor tissues were established, but no cell line was successfully generated from *Stc1*^*+/+*^ tumor tissues (Table S1). The three *Stc1*^*+/−*^ tumor cell lines were used for *Stc1* gene expression analysis (Figure 3B).

#### Human samples

Human lung adenocarcinoma sections of formalin-fixed paraffin embedded samples were obtained from the Pathology Department of the University Hospitals of Leicester NHS Trust and collected for research purposes under ethical approval: LREC 14/EM/1159 held by Dr John Le Quesne.

### Method Details

#### Flow cytometry

Flow cytometry for cell surface markers was performed as described (Kamata et al., 2015) using fluorochrome (FITC, PE, APC, or AlexaFluor®488)-conjugated or biotinylated antibodies for B220, CD11b, CD11c, CD31, CD4, CD45, CD8a, F4/80, Gr1, CD103, CD197 (CCR7), CD117 (c-Kit), MHC class II (I-A/I-E), CD170 (Siglec-F), CD326 and Sca1 for primary staining, and streptavidin-APC (eBioscience) for secondary staining for biotinylated primary antibodies. For SPC intracellular staining, CD45-stained lung cells were fixed/permeabilized using BD Cytofix/Cytoperm™ kit (BD Biosciences) according to manufacturer’s instructions, re-suspended in PBS, and frozen at −20°C for 24 hours. Then the frozen cells were thawed in a 37°C water bath, and stained with anti-SPC antibody (FL-197, Santa Cruz #sc-13979) in BD Perm/Wash™ buffer (BD Biosciences) at 37°C for 45 min, followed by AlexaFluor®488-conjugated anti-rabbit antibody (ThermoFisher Scientific) staining in BD Perm/Wash™ buffer at room temperature for 20min. The stained cells were quantified using BD FACScanto II flow cytometer (BD Biosciences). Flow cytometry detection of dead cells stained with Fixable Viability Dye eFluor™ 780 (Thermo Fisher Scientific) was performed according to the manufacturer’s instructions. BrdU uptake by TAFs/IMCs was analyzed using FITC BrdU Flow Kit (BD Biosciences) according to manufacturer’s instructions.

#### Immunohistochemistry (IHC) staining

IHC staining was performed on paraformaldehyde (PFA)-fixed, paraffin-embedded mouse lung sections for αSMA and F4/80. Rehydrated lung sections were boiled in Tris (10 mM) / EDTA (1 mM) buffer (pH9) for 10 min and blocked with 5% BSA for 30 min followed by incubation with specific blocking solutions included in the polymer detection kits below. Primary antibody staining was performed with F4/80 (clone CI:A3-1, BioRad) and αSMA (Abcam #ab5694) antibodies for 1hr at room temperature. αSMA staining was detected using Novolink Polymer Detection System (Leica Biosystems), whereas F4/80 staining was developed using ImmPRESS™ HRP anti-Rat IgG (mouse-adsorbed) Polymer Detection Kit (Vector Laboratories) and DAB Peroxidase Substrate Kit (Vector Laboratories), according to manufacturers’ instructions.

#### Immunofluorescence (IF) staining

IF staining of primary cultures of lung tissues was performed essentially as described (Kamata et al., 2015). For E-cadherin/vimentin dual staining, E-cadherin-stained cells were blocked for endogenous biotin (15min treatment with streptavidin solution followed by 30 min incubation in PBS containing 0.5 mg/ml biotin) and stained with vimentin antibody (clone LN-6, Sigma #V2258). Then the cells were incubated with biotinylated anti-mouse IgM antibody (clone II/41, eBioscience) followed by AlexaFluor®488-conjugated streptavidin (ThermoFisher Scientific) staining. For cell surface GRP94 immunofluorescence staining, live cells (adhered to coverslips) were first stained with GRP94 antibody (clone 9G10, Enzo Life Sciences #ADI-SPA-850) on ice to inhibit internalisation of antibody-bound cell surface GRP94, followed by AlexaFluor®568-conjugated anti-rat secondary antibody (ThermoFisher Scientific) staining on ice. Then the stained cells were fixed in 4%PFA/PBS for 10min and permeabilised in 0.4% Triton X-100/PBS for 10min for cellular SR-A1 staining followed by AlexaFluor®488-conjugated anti-rabbit secondary antibody. Confocal imaging was performed using an Olympus FV1000 confocal laser scanning system with an inverted IX81 motorised microscope equipped with UPlanSApo 60x/1.35NA objective (Olympus). Obtained images were deconvoluted using Huygens Essential software (Scientific Volume Imaging) and processed using ImageJ software (NIH).

#### Cell purification

Cell purification from BVE lung tumor tissues were performed as described (Kamata et al., 2015). Macrophage-lineage cells from SPK lung tumor tissues were also purified using the same method (Kamata et al., 2015) and 90%–95% purity of CD11c+ cells was routinely achieved (Figure S4). To deplete macrophage-lineage cells from lung tumor tissues, tissues were first digested in RPMI1640 medium containing collagenase/DNase/5% FBS for 1h as described (Kamata et al., 2015) and released single cells were removed by passing through a 70 μm cell strainer. The tissues remaining on cell strainers were recovered and further incubated in the same enzyme buffer for 2h at 37°C. Then, red blood cells were lysed as described (Kamata et al., 2015), and the fully digested tissues were incubated on tissue culture plates for 1h to remove highly-adhesive CD11c+ cells contaminating at this stage. Non-adherent cells after the incubation were collected, and re-plated at 5 × 10^6^ cells/well (12-well plates) in 2 ml/well DMEM/10% FBS. The established primary cultures were serially passaged using trypsin for propagating lung fibroblasts in DMEM/F12 media containing 10% FBS (Figure S3) or subjected to serum-free culture to establish epithelial tumor cell lines (see above, EXPERIMENTAL MODEL AND SUBJECT DETAILS, Primary mouse lung tumor cell lines). Of note, non-immortalized primary lung tumor cells from BVE/SPK models did not survive when plated as single cells by trypsinisation under our culture conditions, whereas passaging by trypsinisation facilitated fibroblast enrichment (Figure S3).

#### Cell culture

Purified macrophage-lineage cells were cultured in serum-free DMEM for 72-96h for SR-A1 inhibition with 10 μM rhein (Sigma) and/or for collecting culture media/cell lysates for ELISA/immunoblotting, or in DMEM/5% FBS for 48-120h for 50% HEK293^T^-CM (Mock or STC1-CM) and/or 75 μg/ml fucoidan (Santa Cruz) treatment to induce macrophage differentiation. Mouse primary lung fibroblasts enriched by serial passaging of lung tissues (Figure S3) were subjected to modified 3T3 culture, in which 3 × 10^5^ cells were re-plated every 4-6 days in DMEM/10% FBS, or co-cultured for 72h with IMCs in serum-free medium for TGFβ1 ELISA or in DMEM/F12 containing 10%FBS ± 1 μM SB431542 (Cambridge Bioscience) for BrdU uptake assay. HEK293^T^ cells were maintained in DMEM/10%FBS.

#### qRT-PCR

RNA extraction was performed using GenElute Mammalian Total RNA Miniprep Kit (Sigma), followed by DNase treatment using DNA-free Kit (Thermo Fisher Scientific), according to the manufacturers’ instructions. qRT-PCR was performed as described (Hey et al., 2016), using primer pairs previously described (Kamata et al., 2015, Nguyen et al., 2009) (Table S4).

#### Immunoblotting and immunoprecipitation

Detergent soluble protein lysates and CM samples were analyzed by immunoblotting as described (Kamata et al., 2015). Primary antibodies used for immunoblotting are listed in the KEY RESOURCES TABLE. Whole cell lysates were prepared by solubilising in 1x SDS sample buffer (62.5mM Tris-HCl (pH6.8), 2% SDS, 10% glycerol, 0.01% bromophenol blue). Proteins insoluble in NP40 lysis buffer (1% IGEPAL® CA-630, 50mM Tris-HCl (pH7.4), 150mM NaCl) were solubilised in 1x SDS sample buffer as “detergent insoluble” protein lysates. Immunoprecipitation of His-tagged STC1 was performed using 5 μg of 6x-His Tag antibody (clone HIS.H8, ThermoFisher Scientific) and Dynabeads Protein G (ThermoFisher Scientific) according to the manufacturer’s instructions.

#### Plasmids

The full length human *STC1* cDNA was first sub-cloned into pLEICS-29 and pLEICS-49 mammalian expression vectors, provided by the Protein Expression Laboratory (PROTEX) at Leicester (https://www2.le.ac.uk/colleges/medbiopsych/facilities-and-services/cbs/protex/available-vectore/details-of-vectors/view) to generate pLEICS29-hSTC1-TEV-EGFP and pLEICS49-hSTC1-TEV-His4/FLAG3 expressing human STC1 tagged with C-terminal EGFP (STC1-GFP) or FLAG® (STC1^FLAG^) through the Tobacco Etch Virus (TEV) protease cleavage sequence. Using the pLEICS29-hSTC1-TEV-EGFP as a template, hSTC1-TEV with C-terminal polyhistidine tag (10xHis) was PCR-amplified, and sub-cloned into the multi-cloning site (HidIII/EcoRV) of pcDNA3.1 (ThermoFisher Scientific) to generate pcDNA3-hSTC1-TEV-His10 expressing human STC1 tagged with C-terminal 10xHis (STC1-His10).

#### Recombinant STC1 treatment

Tagged *STC1* cDNAs (STC1^FLAG^, STC1-His10) were transfected into HEK293^T^ cells using polyethylenimine (PEI, branched 25kD, Sigma). Transfected HEK293^T^ cells were incubated for 72-96 hr in serum-free DMEM without media change to obtain CM containing the recombinant proteins. Mock-CM was obtained from HEK293^T^ cells treated with PEI in the absence of *STC1*-expressing plasmids. Mouse primary cultures were treated in DMEM/5% FBS containing 50% HEK293^T^-CM (Mock or STC1-CM) for 2h to evaluate cellular uptake of extracellular STC1. The cells treated with STC1-His10 CM were trypsinized to digest cell surface-bound proteins, followed by solubilizing in 1x SDS sample buffer to prepare whole cell lysates including mitochondrial proteins whereas those treated with STC1^FLAG^ CM were subjected to IF staining using FLAG-M2 antibody.

#### GFP-Trap® and mass spectrometry

Concentrated CM obtained from HEK293^T^ cells producing STC1-GFP was immunoprecipitated using GFP-Trap® beads (Chromotek) according to the manufacturer’s instructions. Co-immunoprecipitated proteins resolved by SDS-PAGE were identified by mass spectrometry as previously described (Kamata et al., 2015).

#### GRP94 immunodepletion

1 mL serum-free CM from confluent *Stc1*^*−/−*^ IMCs was incubated for 18h at 4°C with 5 μg anti-GRP94 antibody (9G10, Enzo Life Sciences)/100 μL Dynabeads Protein G complex (washed 3 times with 1 mL sterile PBS before use). Then, the beads/antibody complex was magnetically removed, and cleared CM was further incubated with 50 μL Dynabeads Protein G for 90min at room temperature, followed by magnetic removal of the beads, to obtain immunodepleted CM. Primary macrophage-lineage cells plated at a low density were cultured for 72h in serum-free DMEM containing 50% immunodepleted CM for flow cytometry analysis of macrophage differentiation. For GRP94 immunodepletion from HEK293^T^ (mock) CM, HEK293^T^ cells were maintained in serum-free DMEM throughout the transfection procedure to ensure complete removal of FBS from the CM. 0.5ml of serum-free mock-CM was incubated for 18h at 4°C with 10 μg anti-GRP94 antibody/100 μL Dynabeads Protein G complex prepared as above. Then, the beads/antibody complex was magnetically removed, and cleared CM was further incubated with 100 μL Dynabeads Protein G for 2h at room temperature followed by magnetic removal of the beads to obtain immunodepleted CM. Primary macrophage-lineage cells were cultured for 120h in DMEM containing 50% immunodepleted CM and 5% FBS for flow cytometry analysis of macrophage differentiation.

#### Enzyme-linked immunosorbent assay (ELISA)

TGFβ1 levels in culture media were quantified using a TGFβ1 Human/Mouse Uncoated ELISA Kit (ThermoFisher Scientific) according to the manufacturer’s instructions. *In vitro* GRP94 binding to surface-immobilized SR-A1 was quantitated using a functional ELISA as previously reported (Raycroft et al., 2012) with minor modifications. Briefly, high-binding 96-well MaxiSorp™ plates (Nunc) were coated with 1 μg/ml recombinant human SR-A1 (R&D Systems) at 4°C for 24h, followed by 1h blocking with 1x ELISA/ELISPOT diluent (ThermoFisher Scientific). Then 25-100 ng/ml recombinant human GRP94 (RayBiotech) were added with 100 ng/ml recombinant human STC1 (ProSpec) for 2h. Plates were then incubated with anti-GRP94 rat monoclonal antibody (9G10, Enzo Life Sciences, 2 μg/ml in 1x ELISA/ELISPOT diluent) for 1h, followed by incubation with ImmPRESS™ HRP Anti-Rat IgG (Vector Laboratories, 1:10 dilution in 1x ELISA/ELISPOT diluent) for 30min. HRP enzyme activity was visualized by 10 min incubation with 1xTMB ELISA substrate solution (ThermoFisher Scientific). For ELISA quantification of cellular uptake of extracellular GRP94, cells treated with 0.67 μg/ml recombinant human GRP94 tagged with 6xHis (RayBiotech) for 2hr at 37°C in the absence or presence of 3.3 μg/ml recombinant human STC1 (ProSpec) were lysed in 0.2ml NP40 lysis buffer. 50 μL of the lysate was applied into high-binding 96-well MaxiSorp™ plates coated with 4 μg/ml anti-His-Tag antibody (clone His.H8, ThermoFisher Scientific) for 24h at 4°C and blocked in 1x ELISA/ELISPOT diluent (ThermoFisher Scientific) for 1h. After 2h incubation of the lysates, GRP94 bound to the His-Tag antibody on the plates were quantified as described for the functional ELISA above. Lysates from the cells not treated with recombinant GRP94 were used as negative controls to confirm that un-tagged endogenous GRP94 was undetectable in this assay condition. The fraction of exogenous GRP94 protein bound to/taken up was calculated according to the formula: [exogenous GRP94 concentration (ng/ml, measured by ELISA) x 0.2 (ml, volume of the lysate)] / [670 (ng/ml, concentration of exogenous GRP94 added to the culture) x culture volume (ml)].

#### STC1 *in situ* hybridization (ISH)

Human *STC1*-ISH was performed using RNAscope® Multiplex Fluorescence Kit v2 (Advanced Cell Diagnostics) with Hs-*STC1* probe (Advanced Cell Diagnostics, #472691) and OPAL-570 tyramide-fluorescent dye (Perkin Elmer), according to the manufacturers’ instructions. For ISH/IF combination staining, lung tissue sections were first stained for ISH, and incubated in blocking buffer (0.1M Tris-HCl (pH7.4), 0.15M NaCl, 0.05% (w/v) Tween 20, 5% BSA) for 30 min at room temperature. The blocked sections were stained with anti-pan-cytokeratin (clone AE1/AE3 from Abcam, 1:1000), anti-CD68 (clone PG-M1 from DAKO, 1:1000), or anti-αSMA antibody (Abcam #ab5694, 1:500) in blocking buffer at 4°C for 18h, followed by secondary staining using SignalStain® Boost IHC Detection reagent (HRP, mouse) (Cell Signaling Technology) or Novolink Polymer Detection System (Leica Biosystems) with OPAL-520 tyramide-fluorescent dye (Perkin Elmer), according to the manufacturers’ instructions. Image acquisition and analyses were performed as described for IF.

#### Database analysis

The genes co-expressed with *STC1* in human lung adenocarcinoma were analyzed through the web interface SEEK (http://seek.princeton.edu/) (Zhu et al., 2015). TCGA lung adenocarcinoma datasets were accessed through cBioPortal (http://www.cbioportal.org/) (Cerami et al., 2012, Gao et al., 2013). Lung adenocarcinoma samples from the TCGA PanCancer atlas dataset were used for Kaplan-Meier survival analysis. Relative contribution of each subset (M0, M1, M2) of macrophages in the TCGA lung adenocarcinoma samples (Cancer Genome Atlas Research, 2014) was estimated using CIBERSORT (https://cibersort.stanford.edu/) (Newman et al., 2015) at a P value threshold of 0.05. Macrophage immatureness defined by M0/M1 and M0/M2 macrophage ratios in *STC1*^high/low^ groups (top/bottom 20 percentile of *STC1* RNaseq gene expression) and survival differences according to M0 and M2 macrophage abundance were evaluated using this deconvoluted dataset. Association of the *STC1* co-expressed genes with overall survival in lung adenocarcinoma patients was assessed using Kaplan-Meier Plotter; Pan-cancer RNaseq (kmplot.com/analysis/index.php?p=service&cancer=pancancer_rnaseq) (Gyorffy et al., 2013). Hazard ratios and 95% confidence intervals were obtained by comparing groups with above/below-median expression for each gene.

### Quantification and Statistical Analysis

Data were represented as mean ± s.d. with each replicate plotted when applicable. Differences between two groups were examined by Student’s t test unless otherwise stated. Mann-Whitney U test was used for comparisons of data deviated from a normal distribution (judged by Kolmogorov-Smirnov tests, p < 0.05). Kaplan-Meier survival analysis was performed using Stata 16 software (StataCorp), and log-rank tests were used to evaluate differences in survival time between two groups.

## Supplemental Information

Document S1. Figures S1–S7 and Tables S1–S4

Document S2. Article plus Supplemental Information
