# Supplementary material for: Fibroblast-Derived STC-1 Modulates Tumor-Associated Macrophages and Lung Adenocarcinoma Development
Source: Cell Rep. 2020 Jun 23;31(12):107802. doi: 10.1016/j.celrep.2020.107802 (PMC7326292; doi:10.1016/j.celrep.2020.107802)
Supplement: Document S1. Figures S1–S7 and Tables S1–S4 [file mmc1.pdf]

Cell Reports, Volume *31*

Supplemental Information

Fibroblast-Derived STC-1 Modulates Tumor-Associated Macrophages and Lung Adenocarcinoma Development

Tamihiro Kamata, Tsz Y. So, Qasim Ahmed, Susan Giblett, Bipin Patel, Jinli Luo, Roger Reddel, and Catrin Pritchard

Supplementary Information

Table S1. Summary of spontaneous immortalisation culture of SPCCre/KRAS^{G12D} lung tissues, Related to Figure 3 and STAR Methods.

Table S2. TCGA and GEO datasets used for SEEK analysis (ranked by cross-validation – based weighting according to the SEEK algorithm), Related to Figure 7 and STAR Methods.

Table S3. The top 100 genes co-expressed with *STC1* in human lung adenocarcinoma identified by SEEK analysis, Related to Figure 7.

Table S4. PCR primers used in this study, Related to Figure 3 and STAR Methods

Figure S1. Gating strategies for flow cytometry analysis, Related to Figures 1 and 2.

Figure S2. Characterisation of lung tumour-associated CD11c+ cells, Related to Figures 1 and 2.

Figure S3. Characterisation of lung fibroblasts derived from BVE lung, Related to Figure 3.

Figure S4. Cell fractionation from the ^{G12D}KRAS-driven lung tumour model, Related to Figure 3.

Figure S5. Characterisation of the GRP94/SR-A1 axis in IMCs, Related to Figure 5.

Figure S6. Chemical inhibition of SR-A1 and TGFβR1, Related to Figures 5 and 6.

Figure S7. Survival impacts of the top 10 genes co-expressed with STC1 in human lung adenocarcinoma, Related to Figure 7.

Table S1. Summary of spontaneous immortalisation culture of SPCCre/KRAS^{G12D} lung tissues, Related to Figure 3 and STAR Methods

Stc1 genotype	mouse ID	Sex	Lung harvest (days p.i.)	confirmation of immortalization (days in culture)	Use in Figure 3
Stc1-KO	5090	M	344	15d	no
	5259	F	309	failed (120d)	no
	5292	M	315	failed (114d)	no
	5356	F	390	failed (82d)	no
	5357	F	390	failed (82d)	no
Stc1-het	5144	M	363	54d	yes
	5204	F	426	failed (89d)	no
	5233	F	358	failed (71d)	no
	5241	M	343	failed (86d)	no
	5247	M	309	53d	yes
	5249	M	309	failed (120d)	no
	5065	M	734	failed (79d)	no
	5258	M	475	69d	yes
Stc1-wt	5242	M	310	failed (119d)	no
	5288	M	414	failed (91d)	no
	5297	M	427	failed (88d)	no
	5498	M	436	failed (107d)	no
	5549	F	438	failed (84d)	no
	5564	F	435	failed (80d)	no

Table S2. TCGA and GEO datasets used for SEEK analysis (ranked by cross-validation – based weighting according to the SEEK algorithm), Related to Figure 7 and STAR Methods

Rank	Dataset	Coexpression. Score	Description
1	GSE40419.RNASEQ	0.488427	The transcriptional landscape and mutational profile of lung adenocarcinoma
2	TCGA-55-01.RNASEQ	0.414821	Lung adenocarcinoma
3	TCGA-73-01.RNASEQ	0.321201	Lung adenocarcinoma
4	TCGA-78-01.RNASEQ	0.257677	Lung adenocarcinoma
5	TCGA-64-01.RNASEQ	0.25457	Lung adenocarcinoma
6	TCGA-05-01.RNASEQ	0.237659	Lung adenocarcinoma
7	TCGA-44-01.RNASEQ	0.232943	Lung adenocarcinoma
8	TCGA-50-01.RNASEQ	0.216521	Lung adenocarcinoma
9	TCGA-75-01.RNASEQ	0.179118	Lung adenocarcinoma
10	TCGA-86-01.RNASEQ	0.147012	Lung adenocarcinoma
11	TCGA-49-01.RNASEQ	0.144921	Lung adenocarcinoma
12	TCGA-97-01.RNASEQ	0.126334	Lung adenocarcinoma
13	TCGA-91-01.RNASEQ	0.089627	Lung adenocarcinoma
14	TCGA-69-01.RNASEQ	0.049626	Lung adenocarcinoma
15	GSE27719.GPL570	0.025082	Lung adenocarcinoma invasion and progression
16	TCGA-38-01.RNASEQ	0.004013	Lung adenocarcinoma

Table S3. The top 100 genes co-expressed with *STC1* in human lung adenocarcinoma identified by SEEK analysis, Related to Figure 7

Rank	Gene	Entrez ID	Coexpression Score	P-Value	Description
1	LOXL2	4017	2.3569	0	lysyl oxidase-like 2
2	ADAMTS4	9507	2.3373	1	ADAM metallopeptidase with thrombospondin type 1 motif, 4
3	PLOD2	5352	2.2638	0.0001	procollagen-lysine, 2-oxoglutarate 5-dioxygenase 2
4	ADAMTS5	11096	2.195	0	ADAM metallopeptidase with thrombospondin type 1 motif, 5
5	ANGPTL4	51129	2.1662	0.0001	angiopoietin-like 4
6	STC2	8614	2.1081	0	stanniocalcin 2
7	ANGPT2	285	2.0912	0	angiopoietin 2
8	HIF1A	3091	2.0844	0.0006	hypoxia inducible factor 1, alpha subunit (basic helix-loop-helix transcription factor)
9	GPR97	222487	2.06	0.0005	G protein-coupled receptor 97
10	LDHA	3939	2.054	0.0007	lactate dehydrogenase A
11	CHSY1	22856	2.015	0.0002	chondroitin sulfate synthase 1
12	LOX	4015	1.9644	0.0003	lysyl oxidase
13	ITGA5	3678	1.95	0.0032	integrin, alpha 5 (fibronectin receptor, alpha polypeptide)
14	PFKP	5214	1.9381	0.0001	phosphofructokinase, platelet
15	TMEM158	25907	1.9106	0.0003	transmembrane protein 158 (gene/pseudogene)
16	SPOCK1	6695	1.8637	0.0001	sparc/osteonectin, cwcv and kazal-like domains proteoglycan (testican) 1
17	ESM1	11082	1.8587	0	endothelial cell-specific molecule 1
18	GAPDH	2597	1.8581	0.0005	glyceraldehyde-3-phosphate dehydrogenase
19	FLT1	2321	1.8388	0	fms-related tyrosine kinase 1 (vascular endothelial growth factor/vascular permeability factor receptor)
20	PPAPDC1A	196051	1.8231	0.0001	phosphatidic acid phosphatase type 2 domain containing 1A
21	TNFAIP6	7130	1.82	0.0011	tumor necrosis factor, alpha-induced protein 6
22	IL11	3589	1.8181	0.0017	interleukin 11
23	APCDD1L	164284	1.8038	0.0003	adenomatosis polyposis coli down-regulated 1-like
24	MTHFD2	10797	1.8	0.002	methylenetetrahydrofolate dehydrogenase (NADP+ dependent) 2, methenyltetrahydrofolate cyclohydrolase
25	GFPT2	9945	1.7919	0.0008	glutamine-fructose-6-phosphate transaminase 2
26	MCAM	4162	1.7838	0.0007	melanoma cell adhesion molecule
27	ADM	133	1.78	0.0002	adrenomedullin
28	GUCA1A	2978	1.7713	0.0005	guanylate cyclase activator 1A (retina)
29	SPHK1	8877	1.7694	0.0031	sphingosine kinase 1
30	KIF14	9928	1.76	0.0054	kinesin family member 14
31	XIRP1	165904	1.7556	0	xin actin-binding repeat containing 1
32	COL12A1	1303	1.7537	0.0021	collagen, type XII, alpha 1
33	SULF1	23213	1.7506	0.0019	sulfatase 1
34	SNAI1	6615	1.7463	0.0001	snail homolog 1 (Drosophila)
35	UHRF1	29128	1.745	1	ubiquitin-like with PHD and ring finger domains 1
36	GPC6	10082	1.7444	0.0001	glypican 6
37	CHEK1	1111	1.735	0.0066	checkpoint kinase 1
38	SLC2A1	6513	1.7344	0.0008	solute carrier family 2 (facilitated glucose transporter), member 1

39	CXCR7	57007	1.73	0	chemokine (C-X-C motif) receptor 7
40	SLC2A3	6515	1.7231	0.001	solute carrier family 2 (facilitated glucose transporter), member 3
41	P4HA1	5033	1.7225	0.001	prolyl 4-hydroxylase, alpha polypeptide I
42	GPR4	2828	1.7219	0.0056	G protein-coupled receptor 4
43	IL6	3569	1.72	0.0016	interleukin 6 (interferon, beta 2)
44	IL8	3576	1.7162	0.0027	interleukin 8
45	NID2	22795	1.7138	0.0008	nidogen 2 (osteonidogen)
46	CCRN4L	25819	1.7088	0.0035	CCR4 carbon catabolite repression 4-like (S. cerevisiae)
47	CALU	813	1.7081	0.0103	calumenin
48	VEGFA	7422	1.6962	0.0007	vascular endothelial growth factor A
49	AKAP12	9590	1.6838	0.0007	A kinase (PRKA) anchor protein 12
50	GBE1	2632	1.6812	0.0014	glucan (1,4-alpha-), branching enzyme 1
51	SLC39A14	23516	1.6806	0.0007	solute carrier family 39 (zinc transporter), member 14
52	THBS1	7057	1.6788	0.0037	thrombospondin 1
53	COL5A2	1290	1.6762	0.0043	collagen, type V, alpha 2
54	CCL26	10344	1.6694	0	chemokine (C-C motif) ligand 26
55	YWHAG	7532	1.6638	0.001	tyrosine 3-monooxygenase/tryptophan 5-monooxygenase activation protein, gamma polypeptide
56	EGLN3	112399	1.6619	0	egl nine homolog 3 (C. elegans)
57	NFIL3	4783	1.6575	0.0024	nuclear factor, interleukin 3 regulated
58	FAM72B	653820	1.6547	1	family with sequence similarity 72, member B
59	PGK1	5230	1.6525	0.0065	phosphoglycerate kinase 1
60	PTPRN	5798	1.645	0.0003	protein tyrosine phosphatase, receptor type, N
61	BUB1B	701	1.6431	0.0196	budding uninhibited by benzimidazoles 1 homolog beta (yeast)
62	B4GALT1	2683	1.6381	0.0015	UDP-Gal:betaGlcNAc beta 1,4- galactosyltransferase, polypeptide 1
63	TEAD4	7004	1.6363	0.0016	TEA domain family member 4
64	SMOX	54498	1.6306	0.003	spermine oxidase
65	ANLN	54443	1.6287	0.0147	anillin, actin binding protein
66	NAMPT	10135	1.6275	0.0023	nicotinamide phosphoribosyltransferase
67	POSTN	10631	1.6263	0.0015	periostin, osteoblast specific factor
68	ITGB1	3688	1.6247	0.0005	integrin, beta 1 (fibronectin receptor, beta polypeptide, antigen CD29 includes MDF2, MSK12)
69	SHCBP1	79801	1.6156	0.0065	SHC SH2-domain binding protein 1
70	COL5A3	50509	1.6144	0.0067	collagen, type V, alpha 3
71	DLGAP5	9787	1.6125	0.022	discs, large (Drosophila) homolog-associated protein 5
72	ERO1L	30001	1.6113	0.0053	ERO1-like (S. cerevisiae)
73	ARHGAP11A	9824	1.5975	0.0057	Rho GTPase activating protein 11A
74	ADAM12	8038	1.595	0.0038	ADAM metallopeptidase domain 12
75	OR51E1	143503	1.59	0.0001	olfactory receptor, family 51, subfamily E, member 1
76	COL11A1	1301	1.5869	0.0012	collagen, type XI, alpha 1
77	INHBA	3624	1.5856	0.0023	inhibin, beta A
78	GREM1	26585	1.5856	0.0007	gremlin 1
79	COL1A2	1278	1.585	0.0097	collagen, type I, alpha 2
80	B3GNT5	84002	1.5844	0.0011	UDP-GlcNAc:betaGal beta-1,3-N-acetylglucosaminyltransferase 5
81	PITPNC1	26207	1.5844	0	phosphatidylinositol transfer protein, cytoplasmic 1

82	CREM	1390	1.5831	0.0005	cAMP responsive element modulator
83	VEGFC	7424	1.5781	0.0025	vascular endothelial growth factor C
84	PRR16	51334	1.575	0.0001	proline rich 16
85	C5ORF46	389336	1.5744	0.0002	chromosome 5 open reading frame 46
86	CCNB1	891	1.5738	0.0262	cyclin B1
87	PNP	4860	1.5706	0.0025	purine nucleoside phosphorylase
88	SEC23A	10484	1.5688	0.0373	Sec23 homolog A (S. cerevisiae)
89	KIF4A	24137	1.5619	0.0192	kinesin family member 4A
90	COL4A1	1282	1.56	0.0064	collagen, type IV, alpha 1
91	CASC5	57082	1.5488	0.0136	cancer susceptibility candidate 5
92	PRR11	55771	1.5488	0.0041	proline rich 11
93	FAM83D	81610	1.5475	0.0054	family with sequence similarity 83, member D
94	DIAPH3	81624	1.5462	0.0086	diaphanous homolog 3 (Drosophila)
95	UCK2	7371	1.5444	0.0088	uridine-cytidine kinase 2
96	KIF18B	146909	1.5433	1	kinesin family member 18B
97	CEP55	55165	1.5431	0.0226	centrosomal protein 55kDa
98	TPX2	22974	1.5406	0.0291	TPX2, microtubule-associated, homolog (Xenopus laevis)
99	CENPI	2491	1.54	0.0067	centromere protein I
100	VCAN	1462	1.5394	0.0029	versican

Table S4. PCR primers used in this study, Related to Figure 3 and STAR Methods

Gene		Sequence	Reference
Braf$^{LSL-V600E}$ (genotyping)	Fwd	5′-GCCCAGGCTCTTTATGAGAA-3′	Mercer et al., 2005
	Rev	5′-GCTTGGCTGGACGTAAACTC-3′ (for LSL-V600E)	
	Rev	5′-AGTCAATCATCCACAGAGACCT-3′ (for WT/Lox-V600E)	
KRAS$^{LSL-G12D}$ (genotyping)	Fwd	5′-AGCTAGCCACCATGGCTTGAGTAAGTCTGCA-3′	Andreadi et al., 2012
	Rev	5′-CCTTTACAAGCGCACGCAGATGTAGA-3′	
KRAS$^{Lox-G12D}$ (recombination)	Fwd	5′-TGACACCAGCTTCGGCTTCCT-3′	
	Rev	5′-TCCGAATTCAGTGACTACAGATGTACAGA-3′	
CAGG-CreER^{TM} (genotyping)	Fwd	5'-CTCTAGAGCCTCTGCTAACC-3'	Hayashi and McMahon, 2002
	Rev	5'-CCTGGC-GATCCCTGAACATGTCC-3'	
Stc1^{WT} (genotyping)	Fwd	5'-AAAAGCCAGAGGTGCAAGAA-3'	N/A
	Rev	5'-TGTGATCGGAATTCCTCGAC-3'	
Stc1^{KO} (genotyping)	Fwd	5'-AGCGCACGAGGCGGAACAAA-3'	
	Rev	5'-AGAGAGCCGCTGTGAGGCGT-3'	
Stc1 cDNA (RT-PCR)	Fwd	5′-AAGTCATACAGCAGCCCAATCA-3′	Nguyen et al., 2009
	Rev	5′-CCAGAAGGCTTCGGACAAGTC-3′	

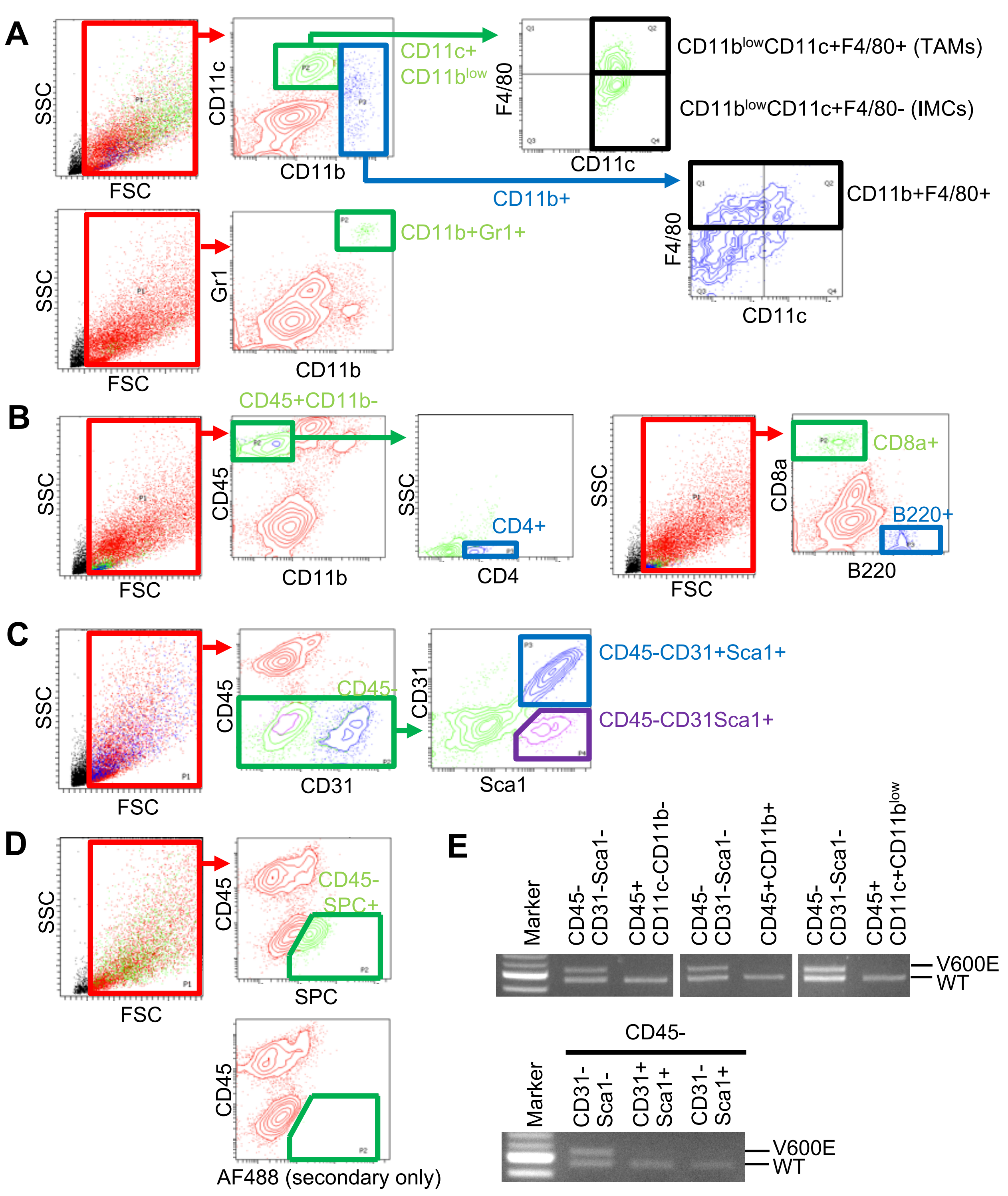

Figure S1. Gating strategies for flow cytometry analysis, Related to Figures 1 and 2. Gating strategies to analyse: **(A)** myeloid populations (CD11b^{low}CD11c+F480+ TAMs, CD11b^{low}CD11c+F480- IMCs, CD11b+F480+ cells, CD11b+Gr1+ cells), **(B)** lymphoid populations (CD4+ T cells, CD8a+ T cells, B220+ B cells), **(C)** endothelial and mesenchymal stromal cell populations (CD45-CD31+Sca1+ cells, CD45-CD31-Sca1+ cells), and **(D)** SPC+ tumour/alveolar type II cells (CD45-/intracellular SPC+ cells). **(E)** No spontaneous recombination of the BRAF^{V600E} allele is detected in the sorted CD45+ myelo-lymphoid populations (top) and CD45- endothelial/mesenchymal stromal cell populations (bottom) from BVE mice. CD45-CD31-Sca1- cells enriched for tumour cells serve as positive controls for PCR detection of the BRAF^{V600E} allele.

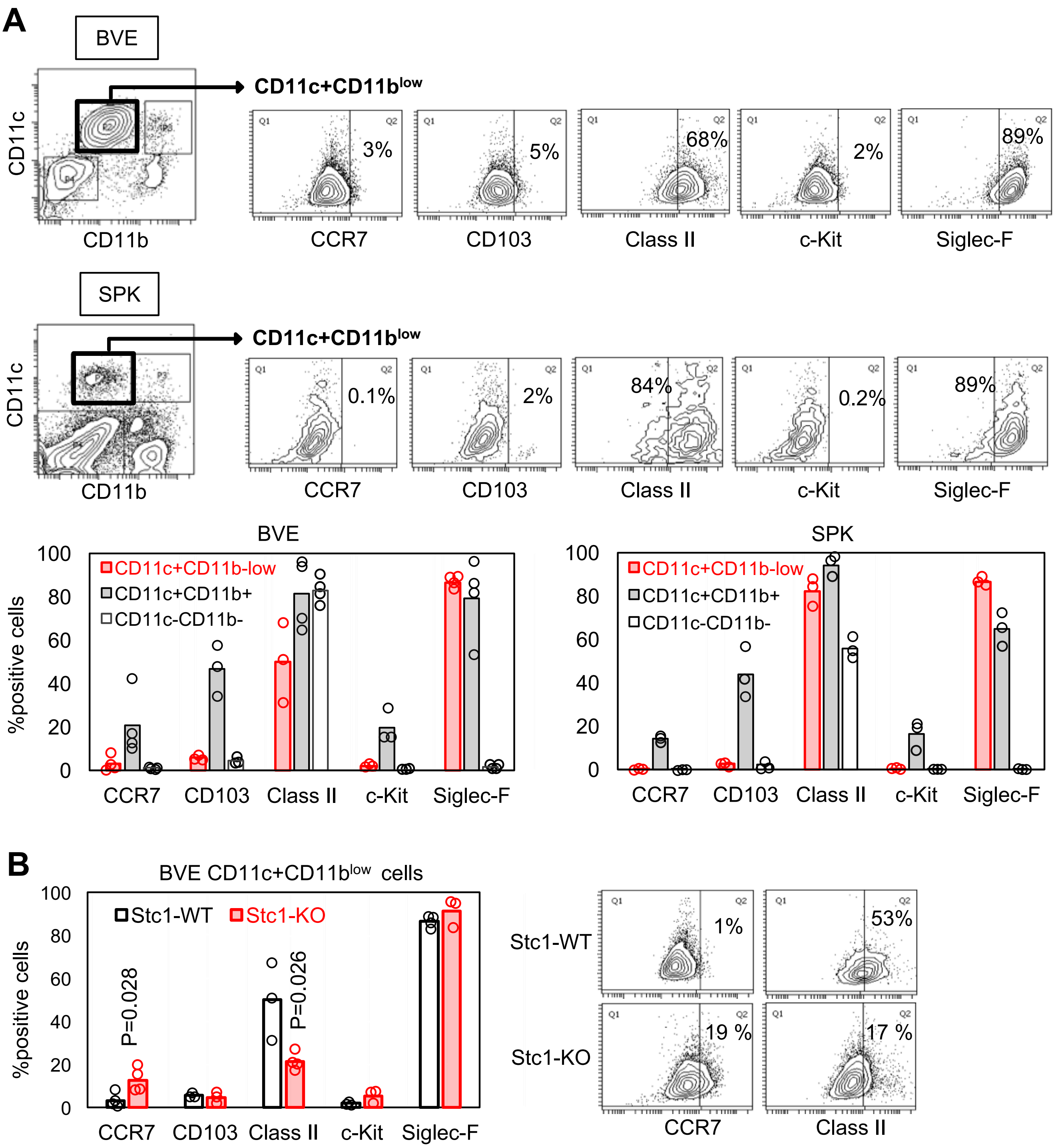

Figure S2. Characterisation of lung tumour-associated CD11c+ cells, Related to Figures 1 and 2. (A) Dendritic cell (DC) and alveolar macrophage (AM) marker expression on CD11c+CD11b^{low}, CD11c+CD11b+ and CD11c-CD11b- cells from Stc1-WT BVE/SPK lungs. Representative flow cytometry plots for CD11c+CD11b^{low} cells (top) and bar graphs summarising n=3-4 (bottom) are presented. **(B)** DC/AM marker expression on CD11c+CD11b^{low} cells from Stc1-WT and Stc1-KO BVE lungs (n=3-4). Representative flow cytometry plots for altered CCR7/MHC class II expression on the CD11c+CD11b^{low} cells from Stc1-KO BVE lung are indicated in the bottom.

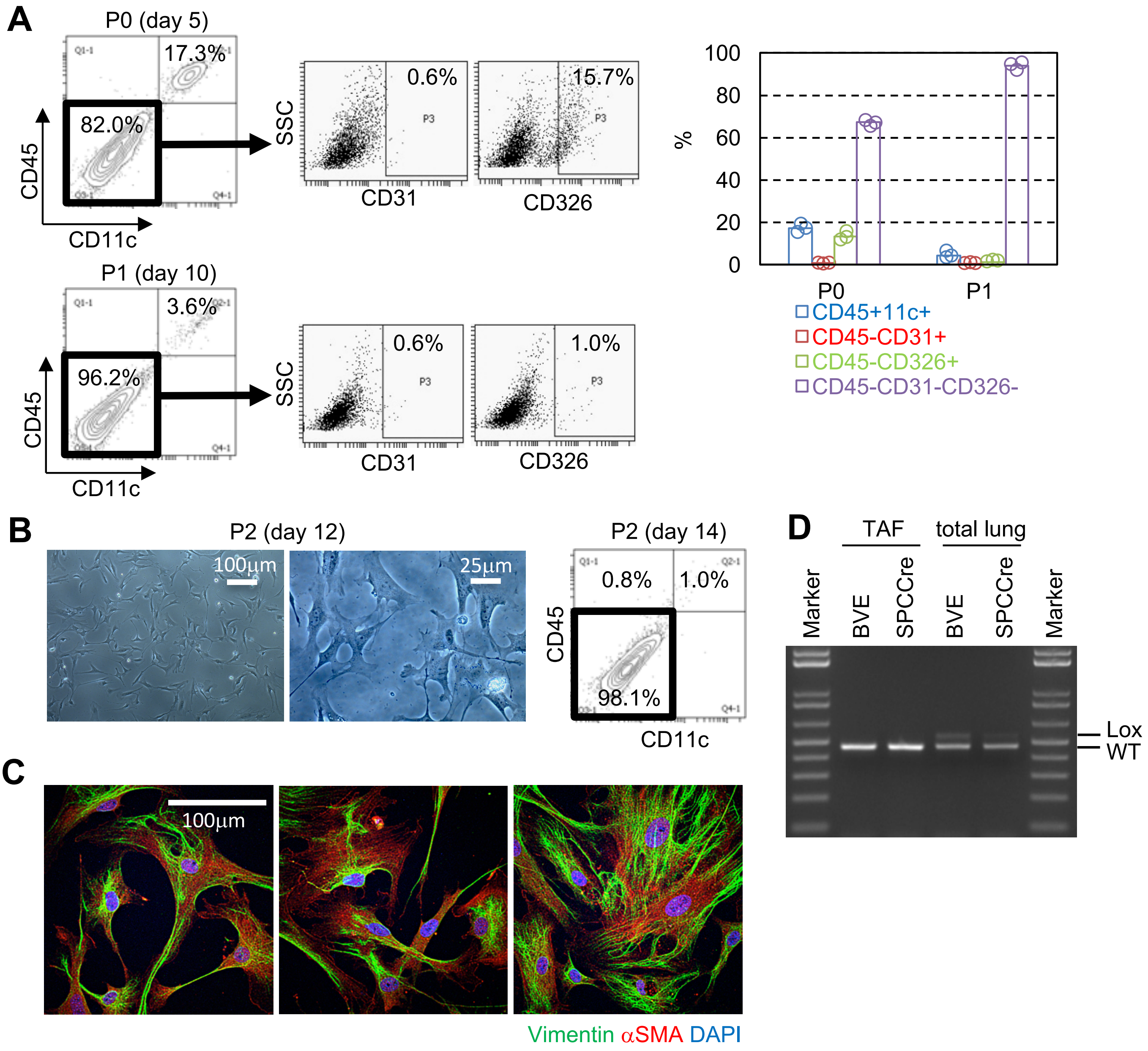

Figure S3. Characterisation of lung fibroblasts derived from BVE lung, Related to Figure 3. (A) Representative flow cytometry plots of BVE lung tissue culture at passage-0 (P0) and passage-1 (P1), demonstrating minimum expression of haematopoietic (CD45/CD11c), endothelial (CD31) and epithelial (CD326) markers at P1. In the right bar graph, %CD45+11c+ IMCs, CD45-CD31+ endothelial cells, CD45-CD326+ epithelial cells are shown (n=3). **(B)** Phase-contrast imaging (left) and flow cytometry analysis (right, CD45/CD11c) of the BVE lung culture at passage-2 (P2), showing fibroblastic morphologies with minimum contamination of CD11c+IMCs. **(C)** Confocal imaging of fibroblastic cells developed in the BVE lung culture at passage-3. Maximum intensity z-projection images of the fibroblastic cells stained for myofibroblast markers, vimentin and αSMA, are shown. **(D)** Braf recombination (Lox allele) was undetectable by PCR of genomic DNA from cultured BVE TAFs. TAFs established from SPCCreBRAF lung serve as a negative control, whereas genomic DNA extracted from whole lung tissues from tumour-bearing BVE and SPCCreBRAF mice was used as a positive control.

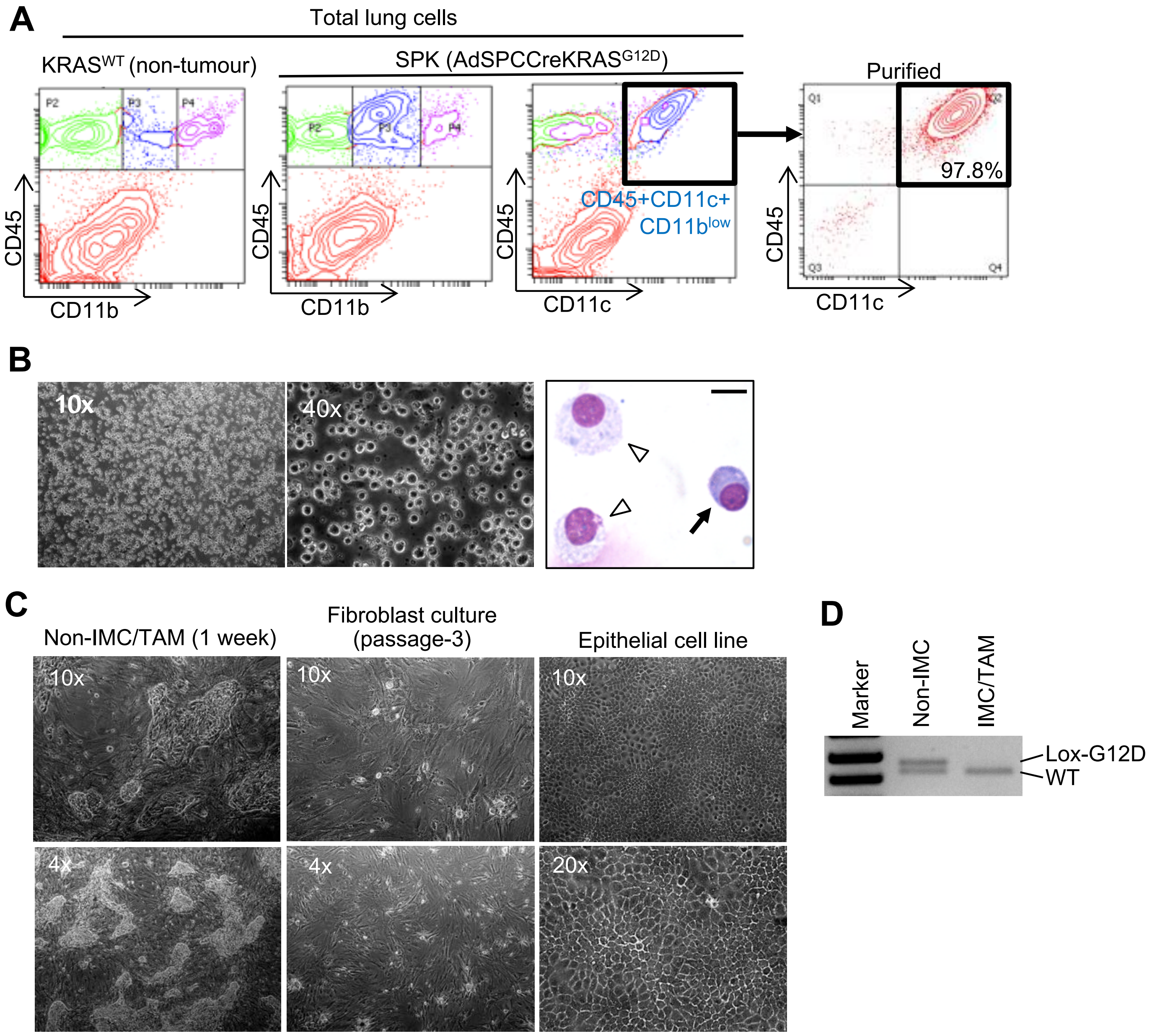

Figure S4. Cell fractionation from the ^{G12D}KRAS-driven lung tumour model, Related to Figure 3. (A) CD45+CD11b^{low}CD11c+ hematopoietic cells expanded in SPK tumour lungs (middle panels, compared to the non-tumour lung, left) were purified and re-assessed by flow cytometry (right panel, showing 98% purity). **(B)** Phase-contrast imaging (left, 10x and 40x objective images) and Giemsa staining of purified CD45+CD11c+ cells (right) showing morphologies consistent with IMC (arrow) and macrophages (open arrow heads). Scale bar = 10µm. **(C)** Representative phase-contrast images of IMC/TAM-depleted (non-IMC/TAM) SPK lung tissue culture at 1 week (left images), fibroblastic cells enriched from non-IMC/TAM culture of SPK lung tissue by serial passages (at passage-3, middle images), and an SPK epithelial tumour cell line (right images). **(D)** Genomic PCR to detect the recombined ^{G12D}KRAS allele (Lox-G12D) in the fractionated cell populations. ^{G12D}KRAS recombination was readily detected in the non-IMC/TAM population (Non-IMC) including tumour cells, but not in the IMC/TAM population (IMC/TAM).

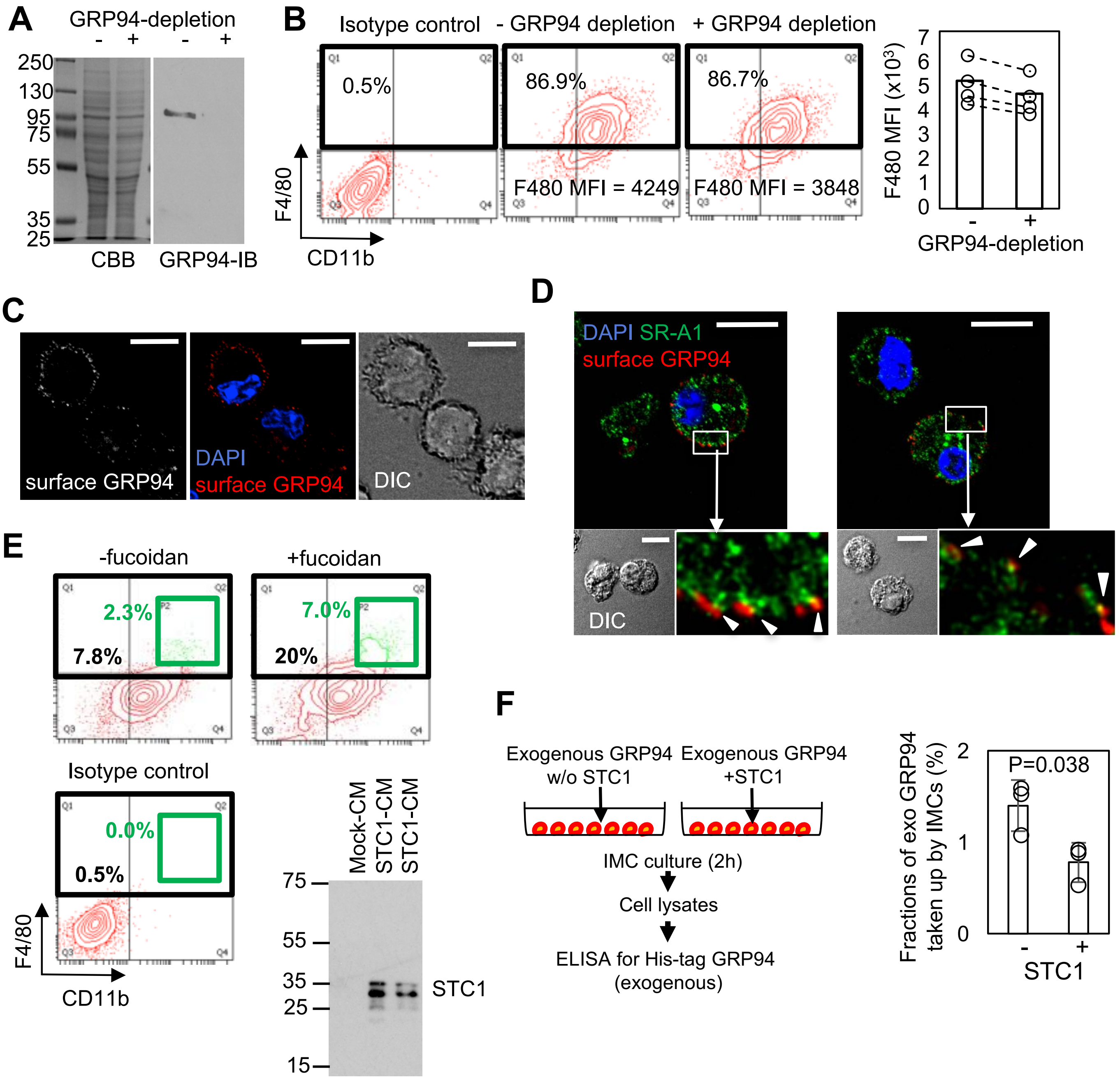

Figure S5. Characterisation of the GRP94/SR-A1 axis in IMCs, Related to Figure 5. (A) GRP94 depletion from HEK293^{T}-conditioned media confirmed by GRP4-immunobloting. Protein loading was monitored by Coomassie Brilliant Blue (CBB) staining. **(B)** F4/80-CD11b flow cytometry plots of IMCs cultured for 5 days with GRP94-depleted HEK293^{T}-CM in the presence of 5%FCS (top panels). A bar graph (right) shows F4/80 mean fluorescence intensity (MFI) on IMCs (right, n=4). **(C and D)** Confocal imaging of cell surface GRP94 IF staining (C) and cell surface GRP94/intracellular SR-A1 dual IF staining (D) of IMCs. Boxed areas are enlarged to show surface GRP94/SR-A1 association (arrowheads). Scale bars = 10μm. Differential interference contrast (DIC) images are shown for morphological identification of the cell surface. **(E)** F4/80-CD11b flow cytometry plots of IMCs cultured for 48hrs +/- 75μg/ml fucoidan in DMEM containing 5%FCS and 50% STC1-CM. %F4/80+ and %F4/80^{high}CD11b^{high} cells are indicated in black and green, respectively. STC1 immunoblotting of STC1-CM used for IMC culture is presented in the lower right. **(F)** (Left) A diagram for the ELISA-based approach to quantitate exogenous, His-tagged GRP94 protein taken up by IMCs. (Right) Fractions of exogenous GRP94 taken up by IMCs, relative to total exogenous GRP94 added into the culture, in the presence or absence of 3.3μg/ml recombinant STC1.

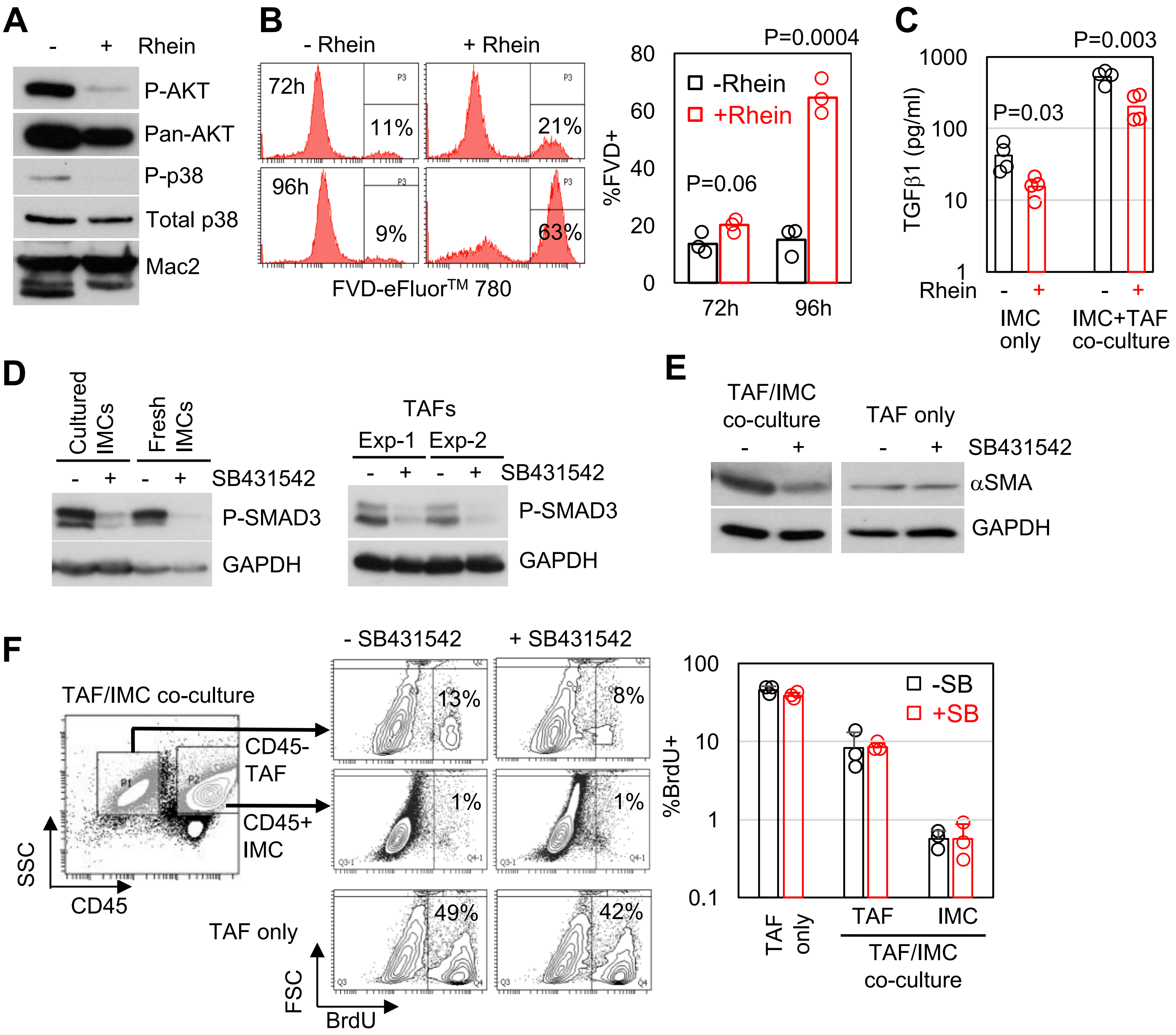

Figure S6. Chemical inhibition of SR-A1 and TGFβR1, related to Figures 5 and 6. (A) AKT/p38 MAPK phosphorylation in IMCs cultured for 72h in serum-free DMEM containing 10μM SR-A1 inhibitor Rhein. **(B)** Viability of IMCs cultured for 72-96h in serum-free DMEM containing 10μM Rhein determined by fixable viability dye (FVD) staining. Representative histograms showing dead cells positively stained for FVD (left), and a bar graph (right) are presented (n=3). **(C)** Rhein suppresses TGFβ1 secretion by IMCs (72h culture in serum-free DMEM with 10μM Rhein) or in TAF/IMC co-culture (72h culture in serum-free DMEM/F12 with 10μM Rhein). TGFβ1 secretion into culture media was quantitated by ELISA. **(D)** Pre-treatment with 1μM SB431542 (TGFβR1 inhibitor, 3h) in serum-free media inhibits TGFβ1 (5ng/ml, 30min)-induced SMAD3 phosphorylation in fresh/cultured (7 days) IMCs (left) and cultured TAFs (right) from BVE lungs. **(E)** SB431542 treatment (1μM, 72h) decreases αSMA expression in TAFs co-cultured with IMCs as evaluated by immunoblotting. **(F)** BrdU uptake by TAFs cultured with or without IMCs. After 48h culture in DMEM/F12 containing 10%FCS +/- 1μM SB431542, cells were labelled with 10μM BrdU for 24h, followed by flow cytometric detection of BrdU+ cells in CD45- TAFs and CD45+ IMCs. The proportion of proliferative (BrdU+) TAFs was robustly decreased by co-culturing with IMCs, but no significant effects of SB431542 on the co-culture-mediated reduction of proliferative TAFs was observed (n=3, bar graph).

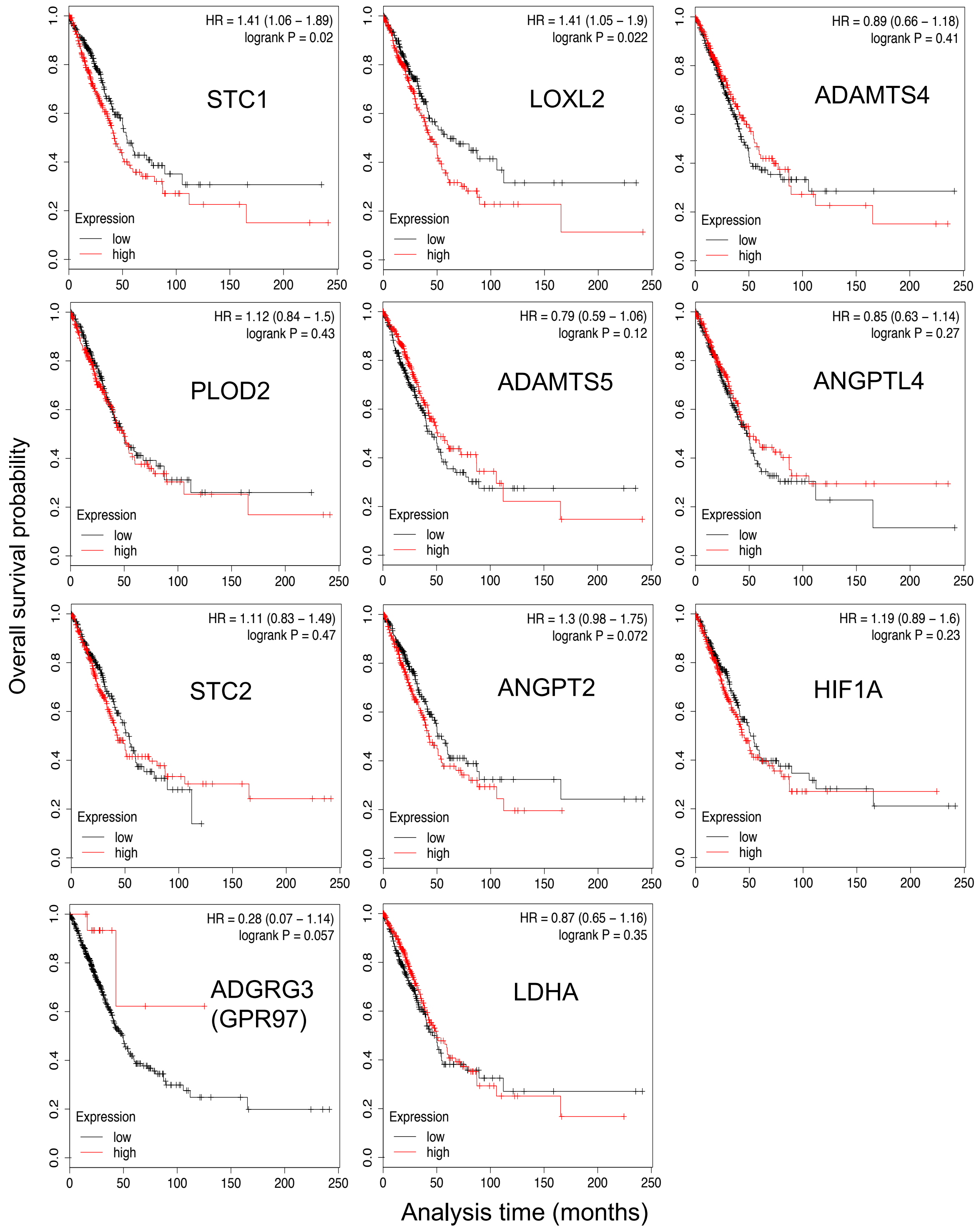

Figure S7. Survival impacts of the top 10 genes co-expressed with STC1 in human lung adenocarcinoma, related to Figure 7. Association of the genes with overall survival in lung adenocarcinoma patients (n=513) was assessed using Kaplan-Meier Plotter (Pan-cancer RNA-seq, https://kmplot.com/analysis/index.php?p=service&cancer=pancancer_rnaseq). Log-rank P-values and hazard ratios with 95% confidence intervals were obtained by comparing samples with above/below-median expression for each gene.
